# Characterization of Signalling Pathways That Link Apoptosis and Autophagy to Cell Death Induced by Estrone Analogues Which Reversibly Depolymerize Microtubules

**DOI:** 10.3390/molecules26030706

**Published:** 2021-01-29

**Authors:** Anne E. Mercier, Renaud Prudent, Michael S. Pepper, Leanne De Koning, Elsie Nolte, Lauralie Peronne, Marcel Nel, Laurence Lafanechère, Anna M. Joubert

**Affiliations:** 1 Department of Physiology, School of Medicine, Faculty of Health Sciences, University of Pretoria, Pretoria 0001, South Africa; elsa07.nolte@gmail.com (E.N.); marcelverwey4@gmail.com (M.N.); laurence.lafanechere@univ-grenoble-alpes.fr (L.L.); annie.joubert@up.ac.za (A.M.J.); 2 Institute for Advanced Biosciences, Team Regulation and Pharmacology of the Cytoskeleton, INSERM U1209, CNRS UMR5309, Université Grenoble Alpes, 38700 Grenoble, France; renaud_prudent@yahoo.fr (R.P.); lauralie.peronne@umontreal.ca (L.P.); 3 Institute for Cellular and Molecular Medicine, Department of Immunology, School of Medicine, SAMRC Extramural Unit for Stem Cell Research and Therapy, Faculty of Health Sciences, University of Pretoria, Pretoria 0001, South Africa; michael.pepper@up.ac.za; 4 RPPA Platform, Institut Curie Centre de Recherche, PSL Research University, Paris 75248, France; leanne.de-koning@curie.fr

**Keywords:** microtubules, reactive oxygen species, apoptosis, autophagy, 2-methoxyestradiol analogues, anti-cancer, mitochondrial membrane potential, cell cycle arrest, p27^Kip1^, JNK

## Abstract

The search for novel anti-cancer compounds which can circumvent chemotherapeutic drug resistance and limit systemic toxicity remains a priority. 2-Ethyl-3-*O*-sulphamoyl-estra-1,3,5(10)15-tetraene-3-ol-17one (ESE-15-one) and 2-ethyl-3-*O*-sulphamoyl-estra-1,3,5(10)16-tetraene (ESE-16) are sulphamoylated 2-methoxyestradiol (2-ME) analogues designed by our research team. Although their cytotoxicity has been demonstrated in vitro, the temporal and mechanistic responses of the initiated intracellular events are yet to be determined. In order to do so, assays investigating the compounds’ effects on microtubules, cell cycle progression, signalling cascades, autophagy and apoptosis were conducted using HeLa cervical- and MDA-MB-231 metastatic breast cancer cells. Both compounds reversibly disrupted microtubule dynamics as an early event by binding to the microtubule colchicine site, which blocked progression through the cell cycle at the G_1_/S- and G_2_/M transitions. This was supported by increased pRB and p27^Kip1^ phosphorylation. Induction of apoptosis with time-dependent signalling involving the p-JNK, Erk1/2 and Akt/mTOR pathways and loss of mitochondrial membrane potential was demonstrated. Inhibition of autophagy attenuated the apoptotic response. In conclusion, the 2-ME analogues induced a time-dependent cross-talk between cell cycle checkpoints, apoptotic signalling and autophagic processes, with an increased reactive oxygen species formation and perturbated microtubule functioning appearing to connect the processes. Subtle differences in the responses were observed between the two compounds and the different cell lines.

## 1. Introduction

Microtubules (MTs) are key components of the cytoskeleton of eukaryotic cells. They are dynamic polymeric filaments composed of α- and β-tubulin heterodimers which have the ability to rapidly polymerize or depolymerize within a structural polarity in order to execute their crucial roles in cell division and other physiological processes [1,2]. Targeted perturbation of these finely tuned mechanisms constitutes a major therapeutic strategy, and microtubule targeting agents (MTAs) have already been part of chemotherapeutic regiments for over three decades [3]. MTAs can be loosely classified into MT-stabilizers such as epothilones and taxanes, and MT-destabilizers such as vinca alkaloids, combretastatin and colchicine [1]. The binding of vinca alkaloids or colchicine prevents the curved-to-straight conformational change of tubulin at the tip of the growing MT necessary for the proper incorporation of new tubulin dimers into the MT lattice [4,5]. Central to MTAs’ intracellular mechanism is the impairment of MT functions, which can have an impact at any stage of the cell cycle and induce programmed cell death. Cellular homeostasis including organelle and protein trafficking via the motor proteins kinesin and dynein may be disrupted during interphase. In addition, defects in mitosis can lead to an arrest of the cell cycle progression at the second gap and mitosis (G_2_/M) junction [6,7]. The latter may be attributed to the spindle assembly checkpoint remaining unsatisfied as kinetochores are unable to bind to dysfunctional mitotic spindles [8]. In all likelihood, it is the combination of the direct action of the drugs on MTs along with possible secondary targets, which trigger signalling cascades to induce apoptosis. Although MTAs are successful anti-cancer drugs, their clinical application is curtailed due to dose-limiting side-effects and the development of drug resistance [9,10].

2-Methoxyestradiol (2-ME) initially attracted attention as a possible alternative MTA. This natural metabolite resulting from sequential hepatic hydroxylation and methylation of 17-β oestradiol exhibited promising anti-tumourigenic properties against a wide range of neoplasms both in vitro and in vivo [8,11]. Additionally, 2-ME preferentially spared non-cancerous cells [12,13]. The anti-proliferative effects of 2-ME were attributed to, at least partially, its ability to abrogate microtubule dynamics by binding to the MT colchicine-binding site, resulting in a G_2_/M phase block [8,14,15,16,17]. Although 2-ME underwent phase I and II clinical trials with Entremed as Panzem®, it was not successfully implemented clinically due to its low oral bioavailability and short half-life in vivo [8,12,18].

**Figure 1:**
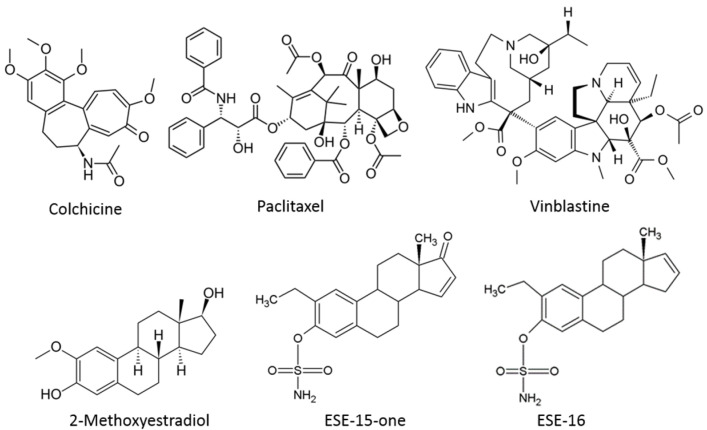
Chemical structures of various microtubule disrupting agents. Colchicine and vinblastine are classical microtubule destabilizers which affect dynamic instability parameters and depolymerize microtubules (concentration dependent). Paclitaxel is an example of a microtubule stabilizing agent. The bottom 3 compounds illustrate the structures of (17 beta)-2-methoxyestra-1,3,5(10)-triene-3,17-diol (2-methoxyestradiol) and the in silico-designed analogues ESE-15-one (2-ethyl-3-*O*-sulphamoyl-estra-1,3,5(10),15-tetraene-3-ol-17-one) and ESE-16 (2-ethyl-3-*O*-sulphamoyl-estra-1,3,5(10)16-tetraene) with their respective substitutions at position C2, C3 and C17 of the parent compound [20].

A range of novel 2-ME analogues have been designed in silico in our laboratory using the Lamarckian genetic algorithm in AutoDockTools4 with the prepare_ligand4py script (Figure 1) [19]. The rationale behind the design was three-fold. Firstly, the aim was to obtain an increased cytotoxicity by C2 and C3 modifications, the efficacy of which has been demonstrated in a number of commercially available cancer cell lines, including the MES-SA/DX5 multidrug resistant sarcoma cells [20]. Secondly, an improved pharmacokinetic profile was envisioned via two mechanisms, namely evasion of the 17β-hydroxysteroid dehydrogenase-mediated metabolism through D-ring modifications, and increasing the binding affinity to erythrocytic carbonic anhydrase II (CAII) to bypass the first-pass hepatic metabolism by addition of a sulpha moiety. Lastly, selective targeting and retention within the tumour was sought by preferential CA IX-binding, an enzyme which is overexpressed in the acidic solid-tumour micro-milieu [21]. Furthermore, polar moieties would allow these compounds to solubilize in better-tolerated aqueous excipients when compared to the 2ME steroidal hydrophobic scaffold [22]. 

The two compounds which fulfilled the desired criteria in silico, namely 2-ethyl-3*-O*-sulphamoyl-estra-1,3,5(10),15-tetraene-3-ol-17-one (ESE-15-one) and 2-ethyl-3-*O*- sulphamoyl-estra-1,3,5(10)16-tetraene (ESE-16) were synthesized and screened with in vitro assays [19]. Promising results yielded reports of IG_50_ (i.e., concentration that inhibits 50% of the cell growth) in the nanomolar range for both these novel compounds in various cell lines, irrespective of oestrogen-receptor status [19,23,24]. This concentration range is substantially lower than that of 2-ME (IG_50_ between 1 and 5 µM) [25]. Both of these compounds displayed the capacity to disrupt microtubule morphology and induce apoptosis via the intrinsic and extrinsic pathways in various cancer cell lines in vitro [19,24,26]. Additionally, an increased number of autophagic vesicles were observed via transmission electron microscopy, together with more intense monodansylcadaverine staining (fluorescent microscopy), increased intracellular autophagy-related light chain 3 (LC3) protein levels and elevated aggresome formation in ESE-16-exposed HeLa cells [24,27]. These findings implicated autophagic processes as part of the drug response in vitro, although the precise role remained hypothetical.

Thus, although certain aspects of the intracellular mechanisms responsible for the oestradiol analogues’ cytotoxic effect have been studied, there are many questions that remain unanswered. Although the in silico approach aimed at designing compounds that bind to the colchicine binding site of the MTs, this has not yet been experimentally confirmed. Moreover, the reversibility of this MT binding in living cells needed to be established prior to any potential further development of these candidate drugs. Most investigations to date have only examined the late cytotoxic outcomes of these compounds, and investigation of the sequential and temporal signalling may illuminate the cross-talk between the various intracellular responses as a consequence of exposure of cancer cells to these molecules. An alteration in MT dynamics can have consequences on the trafficking of intracellular components, including defective autophagic vesicle processing and altered mitochondrial distributions, which could lead to a G_1_/S cell cycle block with the resultant apoptotic signalling. Questions around the autophagic processes induced by ESE-16 remain. It is not known whether autophagy contributes to the cytotoxicity of ESE-15-one and ESE-16 as part of programmed cell death II or whether it confers relative resistance to the treated stressed cells. This research paper reports on experiments performed with the aim of answering these questions.

## 2. Results

### 2.1. ESE-15-one and ESE-16 Effect Microtubules by Binding to the Colchicine Binding Site

We compared the effect of a 2 h exposure to ESE-15-one and ESE-16 on cellular MT to those of colchicine, paclitaxel and vinblastine using immunofluorescence. DMSO, the vehicle, was used as the negative control. The effects on dynamic (tyrosinated) MTs as well the effects on stable (detyrosinated) MTs were analysed [28] in HeLa and MDA-MB-231 cells.

**Figure 2:**
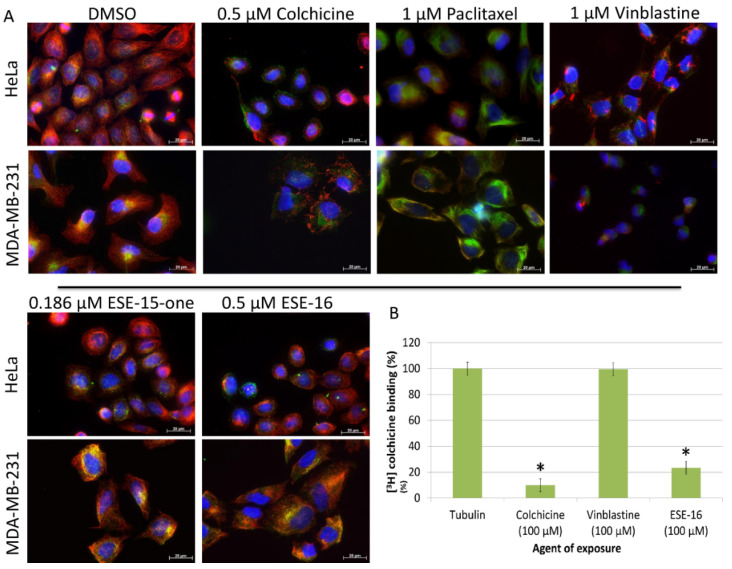
ESE-16 and ESE-15-one affect cellular microtubules by binding to the colchicine-binding site of tubulin. (**A**). Double immunofluorescence of HeLa- and MDA-MB-231 cells exposed to the indicated compounds for 2 h. Dynamic tyrosinated microtubules (MTs) were stained red whereas stable detyrosinated MTs were stained green. Nuclei were counterstained blue with DAPI (63× magnification; scale bar = 20 µm). (**B**). Effect of ESE-16 on the binding of [3H]-colchicine. ESE-16 (100 µM) was used to compete with [3H]-colchicine (50 nM) as described in the methods section. Each value represents the mean ± SEM of 3 independent experiments. Colchicine was used as positive control and vinblastine as negative control. * *p* < 0.05.

As expected, paclitaxel stabilized MTs, as indicated by the enrichment of detyrosinated MTs, while vinblastine caused microtubule depolymerization. When compared to DMSO, ESE-15-one (0.186 µM) and ESE-16 (0.5 µM) both caused MT network perturbations in both HeLa and MDA-MB-231 cells, leaving intact stable pericentriolar MTs enriched with detyrosinated tubulin (Figure 2A). Although these compounds were able to bind to the colchicine binding site of tubulin (ESE-16 competes with the 3[H]-colchicine for its binding on with tubulin (Figure 2B)) their depolymerizing effect was less extensive than that of 0.5 µM of colchicine which induced a complete depolymerization of the MT network. This possibly indicates that these compounds bind to the colchicine binding site on the MTs with less affinity than colchicine.

### 2.2. The Microtubule Depolymerizing Effects of the 2-ME Analogues Are Reversible

**Figure 3:**
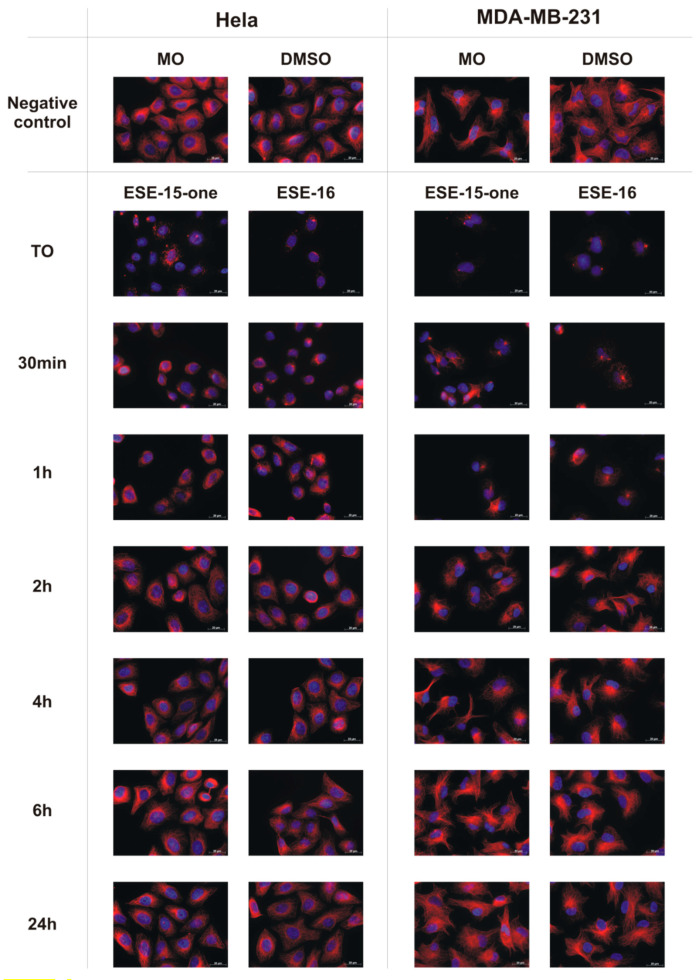
Immunofluorescence analysis of microtubule recovery in Hela and MDA-MB-231 cells after depolymerization with ESE-15-one and ESE-16 (63× magnification). Microtubules are stained red and nuclei are stained blue with DAPI. In both cell lines, full recovery of the microtubule network is observed over a 24 h time period once the molecules had been removed from the medium. Cells exposed to medium only (MO) and DMSO as a vehicle control demonstrate similar intact microtubule networks (Scale bar = 20 µm).

We then investigated whether the effect of the 2-ME analogues on cellular MT were reversible. To achieve this, MDA-MB-231 and HeLa cells were exposed for 2 h to sufficient doses of ESE-15-one and ESE-16 to induce complete MT depolymerization (1.2 µM), as described in the methods section. The medium containing the 2-ME analogues was then removed and replaced with fresh growth medium. The MT networks were then analysed at different time points using immunofluorescence. As shown in Figure 3, complete microtubule depolymerization was observed at T0. The MTs progressively repolymerize after the removal of the compounds, with an almost complete re-polymerization observed after 2 h. Restoration of microtubule function and cell viability was demonstrated by an increase in cell density at 24 h. Results are similar in both cell lines. This indicates that the compounds’ effects on MT are fully reversible.

### 2.3. Kinetics of Cell Death Differ Slightly in Response to ESE-15-one and ESE-16 Exposure

**Figure 4:**
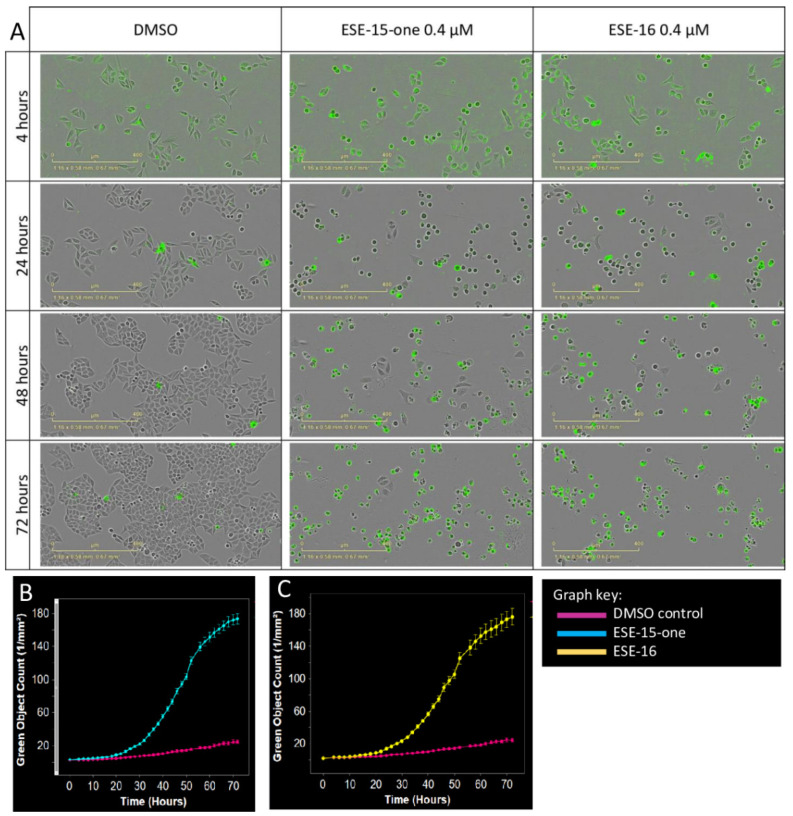
Kinetics of cell death using the real time IncuCyte^®^ Cytotox Green fluorescence assay. HeLa cells were exposed to DMSO (control) or 0.4 µM ESE-15-one and ESE-16. (**A**) Selected time frames of HeLa cells exposed (Scale bars = 400 µm). Graphs indicate a time-dependent increase in green fluorescence (cell death), becoming statistically significant 8 h following exposure to ESE-15-one (**B**) and 12 h following exposure to ESE-16 (**C**).

The kinetics of cell death induced by 0.4 µM ESE-15-one and ESE-16 were compared in Hela cells by 72 h monitoring (Figure 4) using the IncuCyte^®^ live-cell imaging system. When compared to the control (DMSO), phase contrast imaging showed that both compounds induced significant rounding of the cells, which became more pronounced over time. The number of dead cells, indicated by green fluorescence, increased significantly 8 h (4.13 ± 0.49/mm^2^) and 12 h (4.59 ± 0.35/mm^2^) after exposure to ESE-15-one and ESE-16, respectively, compared to DMSO (2.68 ± 0.40/mm^2^ at 8 h and 3.20 ± 0.42/mm^2^ at 12 h). The cytotoxic effect of ESE-15-one and ESE-16 was sustained over the 72-h exposure time, although appearing to plateau at this point. This analysis shows that although both compounds are toxic to cells, there is a slight difference in the kinetics of their effect.

### 2.4. Changes in the Mitochondrial Transmembrane Potential Are Not Detected after a 2-h Drug-Exposure

**Figure 5:**
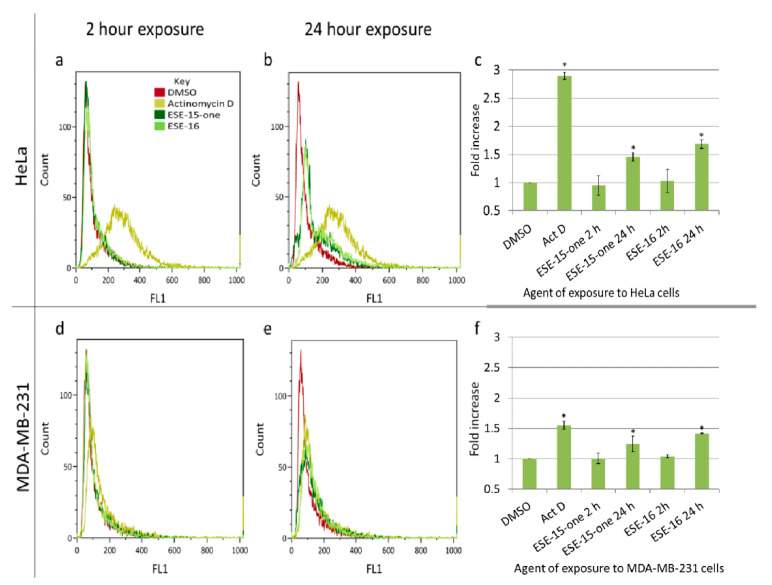
Quantification of the loss in the mitochondrial transmembrane potential induced by ESE-15-one and ESE-16 exposure. Overlay histograms of HeLa (**a**) and MDA-MB-231 (**d**) cells after a 2 h exposure to the compounds. Overlay histograms of HeLa (**b**) and MDA-MB-231 (**e**) cells after a 24 h exposure to the compounds, showing a right shift in the curve indicating the loss of mitochondrial transmembrane potential. The fold-increase in mitochondrial membrane depolarization is summarized in bar charts for HeLa-(**c**) and for MDA-MB-231 cells (**f**). Standard deviation represented by error bars, * *p* < 0.05. The key provided in (a) applies to all the overlay histograms presented.

Microtubule-targeting agents directly and indirectly affect the mitochondria of the exposed cells. Disrupted microtubule dynamics may potentially alter the distribution of these organelles due to impairment of the trafficking function [29]. Secondly, cells undergoing apoptosis lose their mitochondrial transmembrane potential [29], which may be quantified using the cationic Mitocapture™ dye. No significant change in the mitochondrial membrane potentials was detected after a 2-h exposure of both compounds (0.186 µM ESE-15-one and ESE-16 0.5 µM ESE-16) in both cell lines (Figure 5). There was a statistically significant loss of mitochondrial membrane potential after a 24-h exposure in both cell lines with both compounds. ESE-15-one caused a 1.46 ± 0.07 and 1.24 ± 0.13-fold-increase of HeLa- and MDA-MB-231 cell mitochondrial membrane depolarization, respectively. A 1.68 ± 0.08-fold-increase in mitochondrial membrane depolarization was quantified after a 24-h ESE-16 exposure in HeLa cells, and a 1.42 ± 0.01-fold-increase in MDA-MB-231 cells.

### 2.5. Superoxide Production Is Increased by ESE-15-one and ESE-16-Exposure in Both Cell Lines

**Figure 6:**
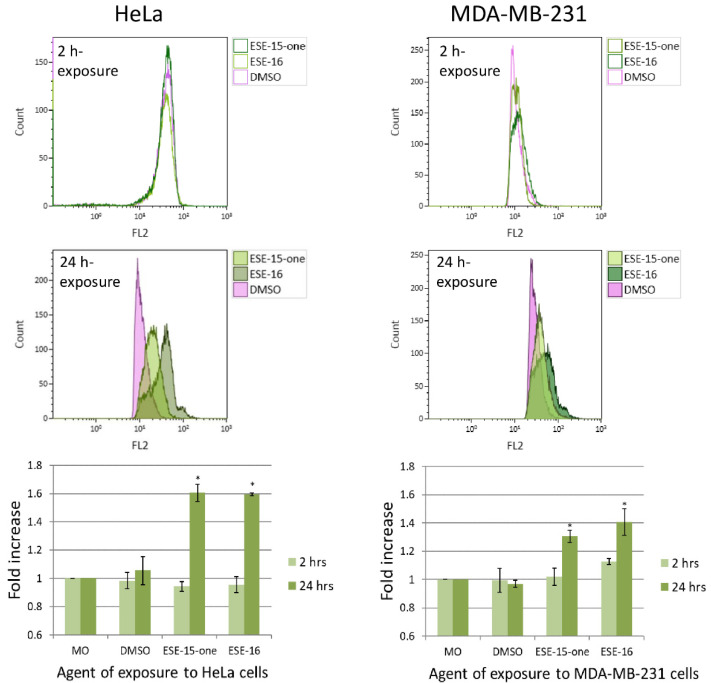
Overlay histograms and bar charts representing the fold-increase in superoxide production in HeLa and MDA-MB-231 cells after a 2- and 24-h ESE-15-one and ESE-16 exposure. Bar charts represent the mean fold-increase over 3 biological repeats, error bars indicate SEM, and *p < 0.05.

Increasing levels of reactive oxygen species (ROS) may induce autophagy either as a means to survive, or to initiate both programmed cell death types I and II if levels exceed a threshold [30,31]. Superoxide, as representative of ROS formation in HeLa- and MDA-MB-231 cells in response to ESE-15-one and ESE-16 exposure for either 2 h or 24 h, was evaluated by quantifying HE oxidation to 2-hydroxy-ethidium using flow cytometry [32]. A 2 h exposure to the compounds did not induce a statistically significant change in superoxide production in either cell line (Figure 6). After 24 h, there was a statistically significant increase in super oxide production in the HeLa cells (1.61 ± 0.06-fold in ESE-15-one- and 1.6 ± 0.01-fold in ESE-16-exposed cells) as well as in the MDA-MB-231 cells (1.31 ± 0.04-fold and 1.41 ± 0.09-fold in ESE-15-one- and ESE-16-exposure respectively).

### 2.6. The 2-ME Analogues Cause a Prolonged Transit through Metaphase and Induced a G_2_/M Block

**Figure 7:**
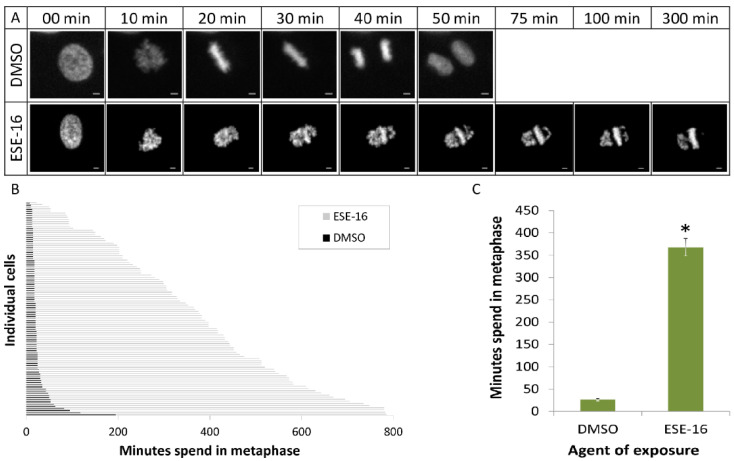
Time-lapse imaging of HeLa Kyoto cells exposed ESE-16. (**A**) Selected frames from time-lapse imaging of HeLa Kyoto cells exposed to 0.4 µM ESE-16 illustrating misaligned chromosomes. Scale bars = 10 µm. (**B**) Analysis of the duration of metaphasis in HeLa Kyoto cells treated with DMSO (control) or with ESE-16, as indicated. Duration of metaphase was analysed from the representative time-lapse imaging experiments shown in A. The data represent 100 cells of each treatment. (**C**) Bars indicate average time spent in metaphase (100 individual cells), with the SEM represented by T-bars, *p < 0.001).

The mitotic progression of fluorescent HeLa Kyoto cells was monitored using time-lapse imaging in order to quantitate the anti-mitotic effect of ESE-16-exposure. Cells exposed to 0.4 µM ESE-16 for 24 h displayed a significant increase in mitotic cells with misaligned chromosomes. The average duration of metaphase increased in cells exposed to ESE-16 (367.85 ± 19.89 min) compared to the DMSO vehicle control (26.3 ± 2.4 min) (Figure 7).

In accordance with the effect of ESE-16 on mitosis, flow cytometry analysis using propidium iodide staining indicated that a 24-h exposure of HeLa and MDA-MB-231 cells to 0.5 μM ESE-16 or 0.186 μM ESE-15-one induced a cell-cycle arrest at the G_2_/M phase. Additionally, there was an increase in apoptosis as indicated by the increased number of cells in the sub-G_1_ phase (Supplementary results Section S2.1).

### 2.7. The Estrone Analogues Increase LC3B Expression and Induce Apoptosis, a Phenomenon Which Is Partially Attenuated if Autophagy Is Blocked

Induction of autophagic processes and apoptotic cell death were further investigated. Annexin V quantification demonstrated a significantly increased number of cells undergoing apoptosis with a concomitant decrease in cell viability after a 24 h-exposure to both drugs, a trend not yet visible after a 2 h-exposure (Supplementary data Section S2.2). Induction of autophagic process was then quantified by measurement of the fluorescence intensity resulting from the intracellular binding of the anti-LC3B/MAP1LC3B antibody conjugated to Alexa Fluor 488. A statistically significant elevation of LC3B in both HeLa and MDA-MB-231 cells after 24 h indicated the possible involvement of autophagy in response to both compounds (Supplementary data Section S2.3). In order to define the possible role of autophagy in the cellular response to ESE-15-one and ESE-16 exposure, autophagy was inhibited with either 3MA or wortmannin prior to drug exposure and apoptosis analysis.

**Figure 8:**
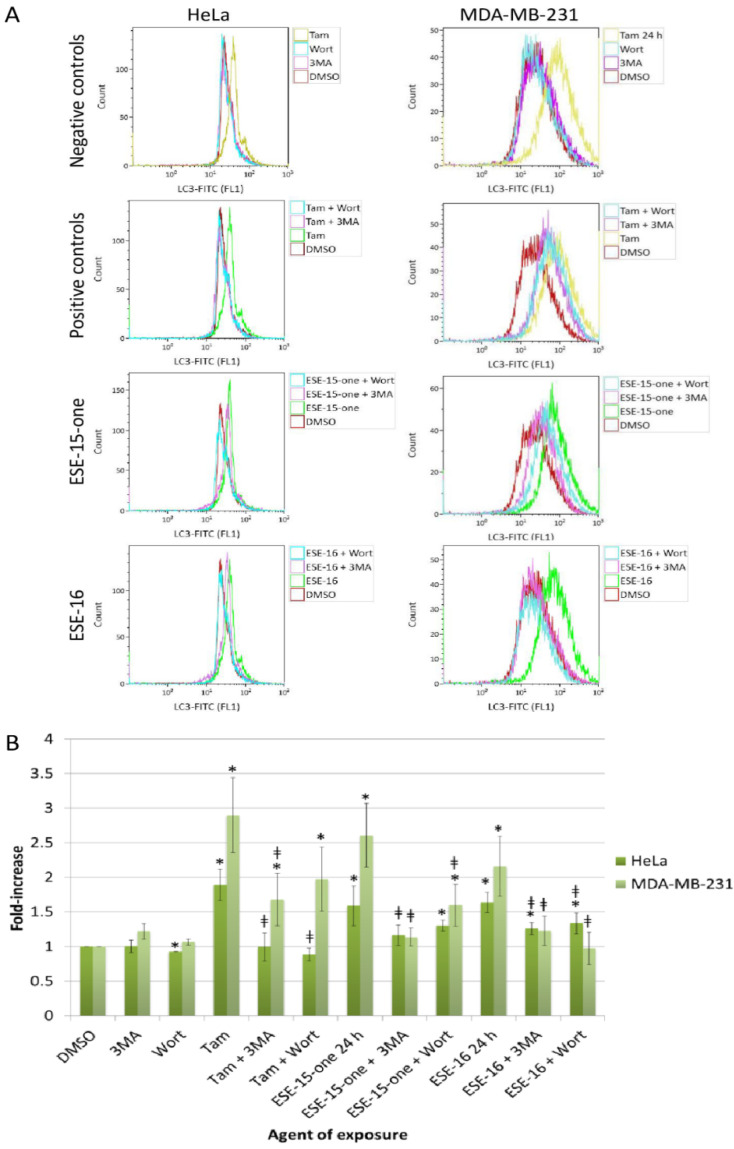
Overlay histograms (**A**) and bar chart (**B**) representing the fold-increase in LC3B detection in HeLa and MDA-MB-231 cells after a 24-h drug-exposure in parallel with autophagy inhibition. Cells were exposed to ESE-15-one and ESE-16 for 24-h either alone, or in combination with 3MA or wortmannin (Wort). LC3B was quantified by capturing fluorescence events of anti-LC3B/MAP1LC3B conjugated to Alexa Fluor 488. A right shift was detected after a 24-h drug-exposure in both cell lines, which was either completely or partially reduced with the addition of 3MA or wortmannin. Tamoxifen (Tam) was used as a positive autophagy control. The standard deviations are represented by error bars. * *p* < 0.05 when compared to the DMSO vehicle control; ^ǂ^ *p* < 0.05 in autophagy-inhibited samples compared to the relevant drug-exposed samples.

No effect on LC3B was demonstrable in both cell lines when exposed to the DMSO, 3MA and wortmannin controls. Tamoxifen was used as a positive autophagy control. The observed increase in LC3B in response to tamoxifen was significantly reduced by addition of 3MA (back to baseline in HeLa cells and significantly reduced in MDA-MB-231 cells) (Figure 8). Although wortmannin inhibition of autophagy in tamoxifen-exposed HeLa cells reduced the LC3B levels to baseline values, MDA-MB-231 samples demonstrated only a decline (not statistically significant. The increase in LC3B detection in HeLa cells after a 24 h ESE-15-one-exposure was reduced significantly from 1.59 ± 0.29-fold to 1.17 ± 0.14-fold by 3MA (complete autophagy inhibition) and to 1.3 ± 0.07 by wortmannin (partial autophagy inhibition). The increase in LC3B in ESE-16-exposed HeLa cells (1.63 ± 0.15-fold-increase) was reduced by the addition of 3MA and wortmannin (incomplete inhibition). ESE-15-one and ESE-16-exposure to MDA-MB-231 cells significantly increased the LC3B levels by 2.61 ± 0.46- and 2.16 ± 0.43-fold, respectively. The addition of 3MA significantly reduced the LC3B levels back to baseline levels, whereas wortmannin addition did the same in the ESE-16 samples. Wortmannin treatment achieved partial inhibition in the ESE-15-one-treated cells (1.6 ± 0.3-fold-increase), although the reduction was significant when compared to the drug-only treated samples.

**Figure 9:**
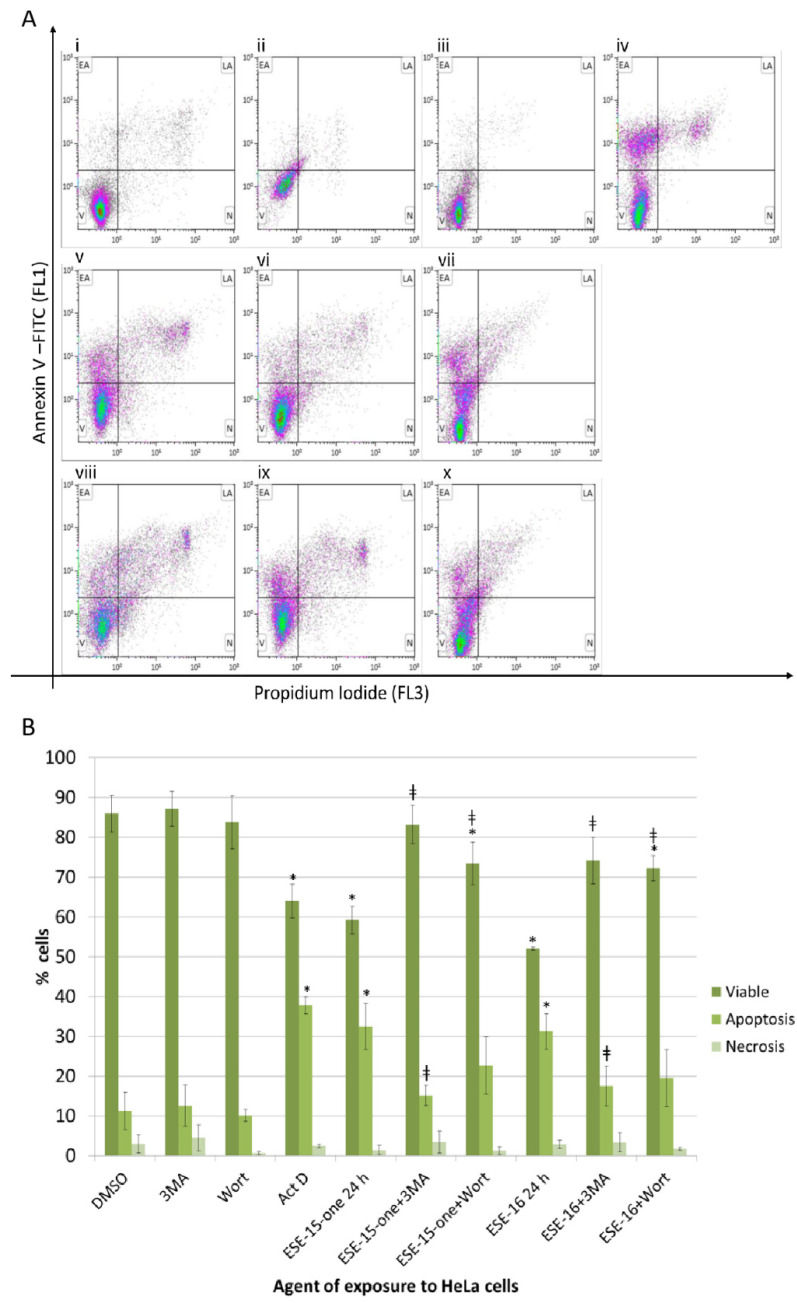
Effect of autophagy inhibition on apoptosis induction in response to the 2ME-analogues. (**A**). Representative Annexin V-FITC scatter plots of HeLa cells exposed to the compounds in the presence or absence of autophagy inhibition. Cells exposed to DMSO (i), 3MA (ii) and wortmannin (iii) were negative controls. Actinomycin D (iv) was the positive apoptosis control. ESE-15-one exposure for 24 h (v) resulted in a decrease in viable cells with a concomitant increase in apoptotic cells. This response was attenuated in the presence of 3MA (vi) and wortmannin (vii). ESE-16-exposure (viii) demonstrated a similar response, again muted in the presence of 3MA (ix) and wortmannin (x). (V = viable cells; EA = early apoptosis; LA = late apoptosis; N = necrosis). (**B**). Graphical representation of the scatterplots. Standard deviation indicated by error bars; * *p* < 0.05 when compared to the DMSO vehicle control; ^ǂ^ *p* < 0.05 when autophagy-inhibited samples were compared to the relevant drug-exposed samples.

In order to understand the effect of autophagy on the compound cytotoxicity, flow cytometric quantification of Annexin V was done on cells in which autophagy is inhibited with 3MA and wortmannin (Figure 9a,b). HeLa cells exposed to ESE-15-one for 24 h demonstrated reduced viability (59.28 ± 3.46%) with a concurrent increase in apoptosis (32.49 ± 5.85%). The addition of 3MA conferred a survival advantage to the cells, as seen by an increase in viable cells and a reduction in apoptotic cells, rendering cell counts similar to baseline levels (DMSO-exposed cell viability was 85.91 ± 0.21% and 11.15 ± 0.21% of the cells were apoptotic). Although wortmannin significantly increased the viable cell population to 73.45 ± 5.3%, the percentage of cells in apoptosis did not significantly decrease when compared to cells exposed to the drug only. ESE-16-exposure to HeLa cells reduced cell viability to 52.03 ± 0.38% and increased apoptosis to 31.24 ± 4.38%. Co-exposure to 3MA partially abrogated this effect, resulting in a viability of 74.14 ± 5.85%, and 17.48 ± 5.01% of the cells in apoptosis, not significantly different to the negative control. Similarly, co-incubation with wortmannin resulted in significantly more viable cells (72.24 ± 3.17%), but no statistical difference in the apoptotic cell population (19.50 ± 7.21%) was detected when compared to the drug-treated samples. Similar results were obtained in MDA-MB-231 cells (Supplementary data Section S2.4).

### 2.8. Signal Transduction Pathways: RPPA and Western Blot Analyses Highlight Signalling Involved in Cell Cycle Progression, Autophagy and Apoptosis

In order to determine the intracellular signalling pathways involved, as well as their temporal engagement, RPPA and Western blots were done at various time-points after the drug-exposure. The RPPA platform was intended to screen for possible pathways influenced by exposure to the 2-ME analogues. Exponentially growing HeLa- and MDA-MB-231 cells were exposed to 0.186 µM ESE-15-one and 0.5 µM ESE-16 in a time series of 0, 0.5, 2, 6, 12, 16, and 24 h. We plotted the normalized expression values over time for each condition (Section S2.5 of the supplementary data) and visually assessed changes. Table 1 summarises the trends in the expression of the various proteins in response to the treatments. Proteins listed at the bottom of the table showed no change in expression or activation when compared to the negative control. The most pronounced changes included sequential increases in phosphorylated p27^Kip1^ and pRb over 24 h. FOXO 1 displayed a decreased expression over 24 h. Phosphorylation of mTOR initially increased from 30 min up until 6 h, and then decreased below that of the standard. Small variations in the cellular responses to ESE-15-one and ESE-16 were observed, as well as differences between the two cell lines investigated.

**Figure 10:**
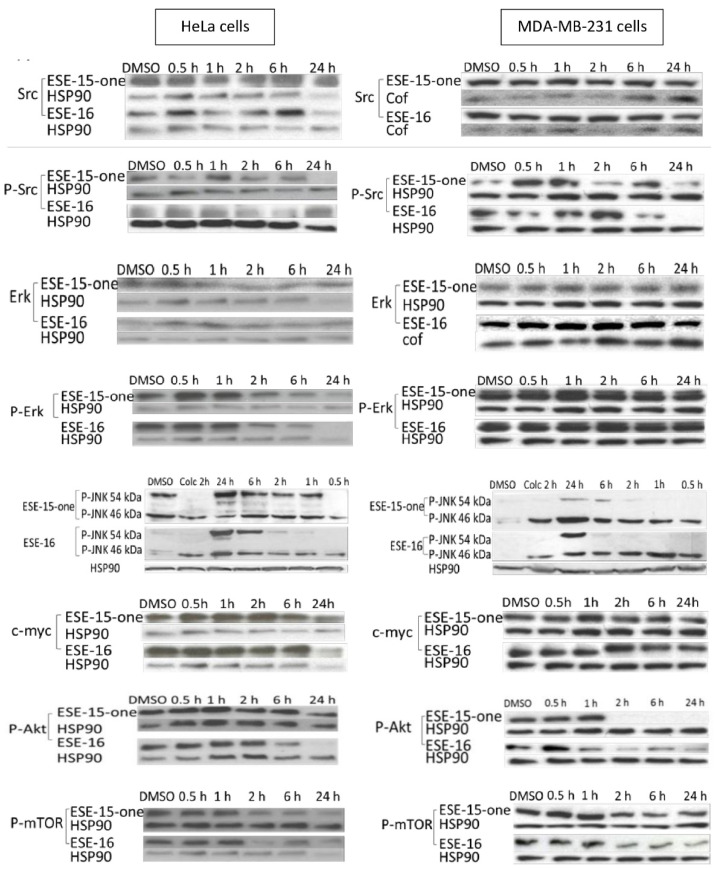
Western blot analysis of the temporal activation of proteins investigated to understand the signalling cascades initiated by ESE-15-one and ESE-16 treatment of HeLa and MDA-MB-231 cells, as indicated.

Western blot analysis was then performed to determine the temporal expression and phosphorylation of selected proteins. Exponentially growing HeLa- and MDA-MB-231 cells were exposed (in a time series) to 0.186 µM ESE-15-one and 0.5 µM ESE-16. To investigate the involvement of the MAPK pathway, and more specifically, the involvement of Erk, its expression and activation by phosphorylation was assessed [33]. HeLa cells displayed no alteration in Erk expression over the 24-h time period, apart from a small decrease at 24 h (Figure 10: supporting data in supplementary results Section S2.7). Both compounds induced an early increase in Erk phosphorylation, which then decreased below baseline levels at 24 h in both cell lines.

JNK (c-Jun *N*-terminal kinase) exists in three isoforms, JNK 1, -2 and -3, the former being distinguished by their size, namely 46 kDa and 54 kDa, respectively [34,35]. HeLa cells exposed to ESE-15-one and ESE-16 displayed incremental phosphorylation of the 54 kDa isoform over 24 h, with the strongest activation at 24 h (Figure 10). The compounds induced phosphorylation of both isoforms, maximally at 24 h, in MDA-MB-231 cells. HeLa cells did not display alteration in c-Myc expression in response to ESE-15-one- and ESE-16-exposure, whereas MDA-MB-231 cells indicated a late decrease induced by both compounds. In HeLa cells, phosphorylation of Akt decreased over time, particularly in response to ESE-16-exposure, which occurred relatively early (2 h). MDA-MB-231 cells initially displayed a brief increase in the phosphorylation of Akt at 30 min in response to both drugs, followed by a decrease in phosphorylation similar to that in HeLa cells. Related to this pathway and due to its role in autophagy, metabolism, survival and proliferation, and actin skeleton regulation, phosphorylation of mTOR in response to the 2-ME analogues was assessed [36]. Both HeLa and MDA-MB-231 cells displayed an early increase in mTOR phosphorylation (between 0.5 and 6 h), which then decreased below baseline levels at 24 h.

## 3. Discussion

The objective of this study was to elucidate the mechanisms and sequential signalling cascades initiated within HeLa and MDA-MB-231 cells after exposure to ESE-15-one and ESE-16 in vitro. A competitive tubulin binding assay confirmed that these compounds did bind to the colchicine binding site on tubulin, but had a diminished depolymerizing effect when compared to colchicine after a 2 h exposure. This indicates that the compounds may either have a decreased affinity to the tubulin, or that they are impeded in crossing the cell-membrane. Moreover, the compounds’ effect on the cellular microtubule network was demonstrated to be reversible, indicating that covalent bond formation between tubulin and the 2-ME derivatives does not occur. Drug reversibility usually enables better control of therapeutic dosing administered during treatment [37]. Furthermore, the ability of cells to recover after withdrawal of the drug may allow normal- and stem cells to restore after treatment in the clinical setting, thereby limiting unwanted long-term side effects [38].

Traditionally, the consequence of MTA perturbation of microtubule dynamics has been synonymous with the induction of cellular metaphase arrest, followed by the induction of programmed cell death [7,10]. In this study, drug-induced metaphase arrest was quantified by time-lapse imaging of HeLa Kyoto cells and in the flow cytometric analysis of cell-cycle progression. The latter experiment also demonstrated an increased proportion of cells in apoptosis, concurrent to a decrease in cell viability after a 24-hour treatment, in both cell lines. However, MTAs may also cause interphase dysfunction that results in a G_1_/S block [39]. In accordance with this concept, an increased p27^Kip1^ and p15^INK4B^ expression was observed in this study, both which are involved in G_1_/S cell cycle progression [40]. Additionally, MTAs may affect many homeostatic mechanisms within interphase cells, such as their ability to correctly traffic components to various subcellular compartments, as well as their ability to move and migrate. 

Autophagy is one such process which requires intact microtubule dynamics for the trafficking of autophagosomes to the lysosomes and degradation of their content after fusion [41,42]. Previously published data indicated that autophagy may play a role in the intracellular response within neoplastic cells exposed to ESE-16 in vitro [24,27,43]. In this study, LC3B quantification via flow cytometry was used as a screening assay based on previously published data correlating an increased level of the protein with an increased number of autophagic vesicles. Both ESE-15-one and ESE-16 exposure resulted in an increased LC3B staining only after 2 h. However, the accumulation of LC3B may be an indicator of increased autophagic induction and/or a decrease in autophagosome clearance, and thus does not assess the complete dynamic process to its conclusion [41,44]. 

Arguing for the up-regulation of autophagy, both RPPA and Western blot analysis indicated the involvement of the mTOR/Akt signalling pathways [45,46] following ESE-15-one and ESE-16 treatment of both cell lines. A small increase in Akt phosphorylation during the initial 2 h of exposure was observed, followed by a significant decrease up to 24-h. This trend was followed precisely by phosphorylation of mTOR. Phosphorylated mTOR suppresses autophagy, whereas the inhibition of the protein, regulated by p-Akt, induces autophagy [46,47]. Additionally, intracellular production of ROS increased sometime after 2 h (clearly significant at the 24 h timepoint), which may link to the induction of autophagy and cross talk between the intrinsic apoptotic pathways.

To establish whether these candidate autophagic processes increase the stress tolerance of the exposed cells or contributed to the cell death processes, autophagy was inhibited with 3MA and wortmannin, after which induction of apoptosis was quantified. Autophagy inhibition (whether partial or complete) reduced cell mortality in both cell lines in response to ESE-15-one and ESE-16 exposure. A study similar in concept was published by Bonet-Ponce et al. (2015) in which ROS generation induced the formation of more autophagosomes due to a reduced clearance evident from the flux assays [48]. Conversely, Viola et al. (2012) published that autophagy inhibition in A549 lung cancer cells increased the cytotoxicity of a novel tubulin inhibitor without involving mitochondrial dysfunction, indicating that these mechanisms involving autophagic functioning are drug- and cell line dependent [49]. Conceptually, studies by Xie et al. (2011) and Qadir et al. (2008) concurred with the latter [50,51].

The hypothesis generated was that a dysfunctional autophagic response, which exacerbates cell death, is triggered by ESE-15-one and ESE-16 treatment in HeLa and MDA-MB-231 cells, together with apoptosis. Potentially, increased ROS-induced autophagy together with a decreased autophagosome turnover results in an accumulation of autophagosomes, which overwhelm the survival process and induce a switch to become programmed cell death type II. Thus, autophagy in this case seems to have an active contribution to cell death induction and augments the cytotoxicity of the compounds under investigation. Further elucidation of molecular events and biological confirmation of this hypothesis would need testing to accept it. In addition, the exact effects which 3MA and wortmannin may have on the cells in the combination treatment would need to be determined (such as induction of p70S6K activity [52]). As inhibitors of phosphoinositide 3-kinase (PI3K), the loss of this signalling cascade may have further reaching consequences than just autophagy inhibition itself [53]. 

As part of the PI3K pathway, the downstream effects of activated Akt activates mTOR, and sequesters forkhead box proteins (FOXO) to the cytoplasm [54]. Various stimuli control FOXO activity by shuttling the protein between subcellular locations, modulating transcription of regulatory domains for cell cycle progression, detoxification of ROS, autophagy and apoptosis [55]. In this study, RPPA analysis indicated a convincing decrease in total cellular FOXO over a 24 h-exposure to ESE-15-one and ESE-16 in both cell lines. Furthermore, caspase-dependent apoptosis induction linked to the negative regulation of FOXO has been described [56,57]. TRAIL inhibition of PI3K/Akt phosphorylation of FOXO in activated hepatic stellate (LX-2) cells enabled nuclear P-FOXO translocation from the cytosol with the consequent induction of apoptosis via the extrinsic pathway [57]. In MDA-MB-231 cells exposed to ESE-15-one and ESE-16, an increase in TRAIL was observed over the 24-h exposure time. The response in HeLa cells was not as prominent. Future investigations would include subcellular fractionation analysis to determine the location of the activated protein in order to define its exact role in the compounds’ cytotoxic effect. 

The ribosomal protein p-p70 S6 kinase (p706Sk) is also under the control of the PI3K-pathway [58]. P-S6 also functions in G_1_/S cell cycle progression [59]. The discordant concept of the current results arises with publications in which rapamycin treatment inhibits translation of proteins essential for G_1_/S cell cycle progression by inhibiting phosphorylation of p70S6k (and even dephosphorylating p70S6), leading to an arrest in cell growth [60,61,62]. The closest answer may possibly come from studying the influenza A virus. Datan et al. (2014) proposed different roles for the two mTOR complexes, mTORC1 and mTORC2 [63], in canonical autophagy induction [64]. Severe infections which resulted in apoptosis induction demonstrated a simultaneous induction of lethal autophagy associated with p-mTOR (Ser2448) suppression, while mTORC1, PI3K and mTORC2 activity increased. P70S6, a substrate of mTORC2, became hyperphosphorylated and its activity (regulated by mTORC1) was required for LC3B-II-formation. Further findings show that mTORC2 locates to mitochondrial membranes where it interacted with and phosphorylated Akt determining cell survival [65,66]. Current RPPA studies indicated phosphorylation of P70S6k after 12 h, together with a transient early increase in p-mTOR and PI3K in MDA-MB-231 cells. However, any correlation to the above hypotheses, as well examining the behaviour of the mTOR complexes directly, would need extensive investigation.

JNK phosphorylation may be seen as a signalling adaptor integrating multiple responses of the cell to various stimulants via the mitogen-activated protein kinase (MAPK) pathway. JNK signalling seems to be integral to 2-ME-induced apoptosis via the intrinsic pathway (and in response to increased ROS) [67] and is also sequentially increased over a 24-h exposure to ESE-15-one and ESE-16 in both the cell lines investigated. Multiple myeloma cells exposed to 2-ME induced up-regulation of JNK, with its subsequent translocation to the mitochondria which resulted in mitochondrial outer membrane permeabilization, initiating the cytochrome *c*- second mitochondria-derived activator of caspases (SMAC)-caspase 3 activation [68]. In addition, JNK signalling may mediate a crosstalk between autophagy and apoptosis. Prolonged oxidative and nutritional stress may switch the JNK signalling from pro-survival to pro-apoptotic [69,70]. Extracellular signal-regulated kinase 1/2 (Erk1/2) can mediate signalling to induce autophagy and apoptosis [71,72]. Increased oxidative stress can also induce cell death via JNK, Erk and Src pathways [73]. Erk1/2 activation via the intrinsic pathway has been reported in paclitaxel and cisplatin treated cells. Although previous studies have not linked 2-ME-exposure to increased Erk1/2 activation [74,75], the current study demonstrated a significant increase in p-Erk1/2, starting early (30 min), increasing until the late stages of exposure when it decreased (at 24 h) in MDA-MB-231 cells. This pattern was also seen in Src phosphorylation and TNF-related apoptosis-inducing ligand (TRAIL) expression in MDA-MB-231 cells only. In fact, p-Src was reduced in HeLa cells, indicating that the involvement of Erk1/2 may not be via the Ras/Raf signalling cascade in this cell line. Wong et al. (2010) reported on the induction of non-canonical autophagy and apoptosis by increased ROS formation in various types of cancer cells via Erk1/2 and JNK upregulation [76]. 

When comparing the IG50 values of ESE-15-one and ESE-16 in cancer cells to the concentrations needed for their in vitro inhibition of tubulin assembly [19,24,26], one can conclude that their effects on microtubule dynamics do not solely account for their toxicity. Other pathways and mechanisms are thus expected to contribute to the induction of programmed cell death. For instance, the production of ROS could have genotoxic consequences, inducing apoptosis via this route, independently of the drug’s microtubule effects. Additionally, information about the intracellular drug-metabolism may be useful for a comprehensive conclusion on the signalling pathways induced. The possibility exists that only the intact compounds cause the microtubule abrogation, whereas a metabolite may induce a different signalling pathway via an alternative receptor/interaction. Furthermore, the effect of the abrogated cytoskeleton on autophagosome-lysosome fusion and the subsequent degradation of the vesicular content would need to be assessed to complete the evaluation of autophagic flux in response to treatment with the 2-ME analogues.

## 4. Materials and Methods

### 4.1. Cell Lines, Culture Methods and Chemicals

MDA-MB-231 breast cancer cells (oestrogen- and progesterone receptor-negative) and human cervical adenocarcinoma (HeLa) cells were purchased from the American Tissue Culture Collection (Manassas, VA, USA). HeLa Kyoto cells expressing EGFP-alpha-tubulin and H2B-mcherry were from Cell Lines Service (Eppelheim, Germany). Cells were cultured in Dulbecco’s modified Eagle medium (DMEM) (Gibco^®^) supplemented with 10% heat-inactivated foetal bovine serum (FBS) (Hyclone, Logan, UT, USA), 100 units/mL penicillin and 100 μg/mL streptomycin (Sigma-Aldrich, St. Louis, MO, USA). Phosphate-buffered saline (PBS) was purchased from Gibco^®^. Cells were cultured at 37 °C in a 5% carbon dioxide (CO_2_) humidified atmosphere in a Forma Scientific water-jacketed incubator. All other reagents not specifically mentioned were of analytical grade and purchased from Sigma-Aldrich (St. Louis, MO, USA).

The in silico-designed sulphamoylated 2ME analogues 2-ethyl-3-*O*-sulphamoyl-estra- 1,3,5(10)15-tetraene-3-ol-17-one (ESE-15-one) and 2-ethyl-3-*O*-sulphamoyl-estra-1,3,5(10)16-tetraene (ESE-16), which are not commercially available, were synthesized by Ithemba (PTY) Ltd. Pharmaceuticals (Johannesburg Gauteng, South Africa), dissolved in dimethyl sulfoxide (DMSO) (10 mM stock solution) and stored at −20 °C. Cells were exposed to the experimental molecules at 0.186 µM for ESE-15-one and 0.5 µM for ESE-16 based on previous GI_50_ value determination [20]. Unless otherwise stated, both MDA-MB-231-and HeLa cells (1 × 10^6^) were seeded in 25 cm^2^ flasks and after a 24 h attachment, exposed to 0.186 µM ESE-15-one and 0.5 μM ESE-16 along with the appropriate controls (DMSO vehicle control and positive experimental control) for either 2 h or 24 h. On termination, cells were trypsinized and washed in PBS. For microscopy, 6 × 10^4^ cells were seeded and propagated on 12-mm-round coverslips for 48 h before exposure to the 2-ME analogues.

### 4.2. Tubulin Competitive Binding Assay: Tritiated [3H] Colchicine Binding

The tubulin was prepared from bovine brain as previously described [77]. Pure tubulin (3 µM final concentration) in cold PIPES-EGTA (PME) buffer (100 millimolar (mM) piperazine-*N*,*N*′-bis(2-ethanesulfonic acid) (PIPES), 1 mM magnesium chloride (MgCl2), 1 mM ethylene glycol-bis(β-aminoethyl ether)-*N*,*N*,*N*′,*N*′-tetraacetic acid (EGTA), pH 6.65) was mixed at 4 °C with a mix of [3*H*]-colchicine (82.6 Ci/mmol) (Perkin-Elmer, Courtaboeuf, France, 50 nM final concentration) and the competitor ESE-16 (100 µM final concentration) in a final volume of 200 µL. Following a 30-min incubation at 30 °C, the samples were deposited onto 50 µL of pre-sedimented DEAE Sephadex A25 in BRB80 buffer. All subsequent steps were carried out at 4 °C. Samples were incubated for 10 min with continuous agitation to ensure quantitative binding of tubulin to the gel. Following centrifugation (2400× *g*, 4 min), supernatants were discarded and the pellets containing the bound molecule-tubulin complexes were washed four times with 1 mL of PME buffer. Pellets were incubated for 10 min with 500 µL of ethanol to solubilize the tubulin-bound tritiated colchicine and 400 µL aliquots of the ethanol solutions were transferred to 5 mL of Ultima Gold scintillant (Perkin-Elmer, Courtaboeuf, France) for determination of radioactivity using a liquid scintillation counter (Beckman Coulter, Brea, CA, USA).

### 4.3. Immunofluorescence Microscopy

This method has been described by Paturle-Lafanechère et al. [78]. Cells were exposed to the compounds for 2 h. They were then were permeabilized with OPT buffer (80 mmol/L PIPES, 1 mol/L EGTA 1 mol/L MgCl_2_, 0.5% triton X-100, and 10% glycerol, pH 6.8) at 37 °C and fixed in methanol at −20 °C for 6 min. Cells were incubated in a primary antibody cocktail consisting of L4 rabbit anti-detyrosinated tubulin (Detyr-tubulin, made by Dr Lafanechère) and YL1/2 rat anti-tyrosinated tubulin (the clone YL1/2 was a generous gift from Dr. JV Kilmartin (MRC, Cambridge, UK) to L. Lafanechère [79]) at 1/4,000 in PBS-tween (PBS-T) 0.1% and 0.3% bovine serum albumin. After rinsing in PBS-T 0.1%, cells were incubated with the secondary antibodies Alexa Fluor^®^ 488 anti-rabbit (Invitrogen, Carlsbad, CA, USA), Cy3 anti-rat (Jackson ImmunoResearch Laboratories, West Grove, PA, USA) and 20 µM Hoechst 33342 to stain DNA, in PBS-T 0.1% containing 0.3% bovine serum albumin (BSA). Rinsed slides were mounted and viewed with a Zeiss Axio Imager Z1 microscope controlled by Axiovision software (Carl Zeiss, Oberkochen, Germany) using a X63 oil objective. Images were captured using an Ocra R2 N/B camera (Hamamatsu, Hamamatsu, Japan).

### 4.4. Reversibility of Microtubule Toxicity Effects

Using the above-described protocol [78], MDA-MB-231 and HeLa cells were exposed to a dose range (0.8, 1.0, 1.2 and 1.4 µM) of ESE-15-one and ESE-16 for either 2 or 3 h. An exposure time of 2 h at a drug concentration of 1.2 µM for both compounds induced microtubule depolymerization without visible signs of nuclear distress in either cell lines. These experimental conditions were chosen for the reversibility study. To that aim, the compounds were removed by washing the cells 3 times with PBS (37 °C), before their fixation at various time intervals (0, 30 min, 1 h, 2 h, 4 h, 6 h and 24 h) for immunofluorescence as described above. DMSO was used as a vehicle control at all the time intervals.

### 4.5. Kinetics of Cytotoxicity

The IncuCyte^®^ S3 Live-Cell Analysis System and the IncuCyte^®^ Cytotox Green reagent (Essen BioScience, Hertfordshire, UK), which is a cyanine nucleic acid dye that enables real-time evaluation and quantification of cell death, was used. HeLa cells were seeded into 96-well plates and incubated for 24 h to allow for attachment. Cells were exposed to ESE-15-one and ESE-16, respectively, and the IncuCyte^®^ Cytotox Green reagent (final concentration of 250 nM). The plates were placed in the IncuCyte^®^ S3 Live-Cell Analysis System and two pictures per well were taken every 2 h over a 72-h period.

### 4.6. Changes in the Mitochondrial Transmembrane Potential: Flow Cytometric Quantification Utilizing the MitoCapture^TM^ Mitochondrial Apoptosis Detection Kit

In order to quantify the changes in the mitochondrial transmembrane potential as an early intracellular apoptotic event, the MitoCapture^TM^ mitochondrial apoptosis detection kit (BioVision Inc., Milpitas, CA, USA) was utilized. Actinomycin D (0.1 µL/mL) was used as a positive apoptosis control. On termination, 1 × 10^6^ cells re-suspended in 1 mL diluted MitoCapture™ solution and incubated at 37 °C for 20 min. Cells were centrifuged for 5 min at 1.2× *g* and re-suspended in incubation buffer. Flow cytometry was performed using the FL1 channel (excitation/emission at 488/530 nm), whereas the untreated cells were detected with the FL2 channel (excitation/emission at 488/590 nm) (FC500 system flow cytometer, Beckman Coulter South Africa (Pty) Ltd., Johannesburg, South Africa).

### 4.7. Generation of Reactive Oxygen Species: Flow Cytometric Quantification of Superoxide Molecules

Trypsinised cells (1 × 105) were washed and resuspended in PBS. HE was added to the cells (final concentration of 1 µM), and incubated protected from light (15 min at 37 °C). Superoxide production was quantified with flow cytometry using the FC500 system flow cytometer (Beckman Coulter South Africa (Pty) Ltd., Johannesburg, South Africa) equipped with an air-cooled argon laser excited at 488 nm on the FL2 channel.

### 4.8. Time-Lapse Imaging

HeLa Kyoto cells expressing EGFP-alpha-tubulin and H2B-mcherry were seeded on 2-well glass-slides (Ibidi, Gräfelfing, Germany, #80297) at a density of 7000 cells per well and allowed to grow for 24 h prior to imaging. After treatment with 0.4 µM ESE-16, the slide was placed on a heated stage (37 °C) at 5% CO_2_. Images were acquired every 150 s by a spinning disk confocal laser microscope (Andromeda iMIC) with a focus stabilizer (Field Electron and Ion Company (FEI), Hillsboro, OR, USA). The microscope was equipped with a Plan-Apochromat 10×/0.45 M27 and iXon electron multiplying charge coupled device camera (Andor). Image sequences were acquired using the FEI Live acquisition software v.2.7.0.16 (FEI) and the cellular responses were analysed manually.

### 4.9. Cell Cycle Analysis

Exposed cells were harvested and washed in ice-cold PBS before centrifugation for 5 min at 1.2× *g*. Cells were resuspended in ice-cold PBS containing 0.1% FBS and fixed overnight in ice-cold 70% ethanol. Centrifuged cells were re-suspended in PBS containing propidium iodide (40 μg/mL), RNase A (100 μg/mL) and Triton^®^ X-100 (0.1%). Cells were incubated protected from light at 37 °C for 40 min. Analysis entailed measurement of propidium iodide fluorescence (FL3) on a FC500 system flow cytometer (Beckman Coulter South Africa (Pty) Ltd., Johannesburg, South Africa) equipped with an air-cooled argon laser excited at 488 nm. Aneuploid and aggregated cells, as well as cell debris were gated out by visual inspection. Cell cycle distributions from generated histograms were expressed as percentage of cells in each phase.

### 4.10. Apoptosis Assay

The apoptosis assay was performed using the fluorescein isothiocyanate (FITC) Annexin V apoptosis detection kit with propidium iodide (BioLegend, San Diego, CA, USA) according to manufacturer’s protocol. Cells were collected and re-suspended in 1× binding buffer. Double staining was done by incubating the cell suspension with Annexin V-FITC and propidium iodide solution for 15 min at RT in the dark. Annexin V (FL1) and propidium iodide (FL3) fluorescence were measured with a FACS FC500 System flow cytometer (Beckman Coulter South Africa (Pty) Ltd., Johannesburg, South Africa) equipped with an air-cooled argon laser excited at 488 nm. Results were expressed in percentage of cells in three categories, namely viable cells, apoptotic cells and necrotic cells.

### 4.11. Autophagic Vacuole Quantification

Autophagy-related light chain 3 (LC3) protein determination was used to quantify autophagic vacuoles. Cells were fixed with 0.01% paraformaldehyde in PBS for 10 min, pelleted and re-suspended in ice-cold PBS. Ice-cold methanol was added, and cells incubated for 15 min at 4 °C. Cells were washed twice with cold PBS and incubated in the antibody cocktail (0.5 μg/mL of the rabbit polyclonal anti-LC3B/MAP1LC3B Alexa Fluor 488-conjugated antibody (Novus Biologicals, Littleton, CO, USA), 0.05% Triton X-100, 1% BSA and 40 µg/mL PI) for 2 h at 4 °C in the dark. Cells were washed with washing buffer (PBS/0.05% Triton^®^ X-100/1%BSA) and fluorescence measured with a FC500 System flow cytometer (Beckman Coulter South Africa (Pty) Ltd., Johannesburg, South Africa) on FL1.

### 4.12. Inhibition of Autophagy

The above autophagy and apoptosis studies were done in the presence or absence of agents which inhibit autophagy. Wortmannin (1 µM) or 3 methyladenine (3MA) (5 mM) were added to changed medium after cell adherence. Cells were incubated for 1 h before the addition of the compounds to the experimental samples. 3MA and Wortmannin controls were run in parallel. The above-mentioned Annexin V and LC3 protocols were used to assess the effect of autophagy inhibition on cell death. The results were analysed to ascertain whether the compounds’ cytotoxicity wan enhanced or curtailed when autophagy was inhibited.

### 4.13. Reverse Phase Protein Array Analyses

HeLa and MDA-BM-231 cells (6 × 10^5^) were seeded in 6-well plates and allowed to attach overnight. Logarithmically growing cells were exposed to 0.186 µM ESE-15-one and 0.5 µM ESE-16 for 30 min, 1 h, 2 h, 6 h, 12 h, 18 h and 24 h. DMSO-exposed cells were used as a vehicle control. On termination, cells were washed twice with PBS and boiling laemmli buffer (50 mM Tris (Ph 6.8), 2% sodium dodecyl sulphate (SDS) 5% glycerol, 2.5 mM ethylenediaminetetraacetic acid (EDTA), 2.5 mM ethylene glycol tetraacetic acid (EGTA) and double distilled water (chemicals from Thermo Fisher Scientific, Waltham, MA, USA). Just before use, 50 µl Halt phosphatase cocktail (Thermo Fisher Scientific, Waltham, MA, USA), 1 tablet/5 mL protease inhibitor cocktail (complete MINI EDTA-free from Sigma-Aldrich (St Louis, MO, USA), 4 mM sodium orthovanadate and 20 mM sodium fluoride (Merck Millipore Corp., Darmstadt, Germany) were added to the cells, which were collected by scraping. Samples were boiled at 100 °C for 10 min and passed 5 times though a 25G needle. Samples were ultra-centrifuged at 200,000 rpm for 30 min and the supernatant harvested. A total of 10 µL of the sample was removed for protein quantification, and DTT (final concentration of 2 mM) was added to the remaining samples. Samples were flash-frozen at −80 °C. Protein concentration was determined using the Pierce™ BCA protein assay kit (Thermo Fisher Scientific Inc., Waltham, MA, USA). A standard curve was set up using increasing concentrations of BSA.

Frozen quantified samples were couriered on dry ice to the Institut Curie Centre de Recherche (Paris) where RPPA analyses were performed. Samples were deposited onto Grace Bio-Labs ONCYTE^®^ SuperNOVA^™^, nitrocellulose covered slides (Grace Biolabs, Bend, OR, USA) using a dedicated arrayer (2470 arrayer, Aushon Biosystems, Billerica, MA, USA). Five serial dilutions, ranging from 2000 to 125 µg/mL, and two technical replicates per dilution were printed for each sample. Arrays were labelled with specific antibodies (Table S1 in the supplementary methods) or without primary antibody (negative control), using an Autostainer Plus (Dako, Agilent Technologies, Santa Clara, CA, USA). Arrays were incubated with avidin, biotin and peroxydase blocking reagents (Dako, Agilent Technologies, Santa Clara, CA, USA) before saturation inTris-buffered saline containing 0.1% Tween^®^-20 and 5% BSA (TBS-T-BSA). They were then probed overnight at 4 °C with primary antibodies diluted in TBS-T-BSA. After washes with TBS-T, arrays were probed with horseradish peroxidase-coupled secondary antibodies (Jackson ImmunoResearch Laboratories, Newmarket, UK) diluted in TBS-T-BSA for 1 h at RT. To amplify the signal, slides were incubated with Bio-Rad amplification reagent (Bio-Rad, Watford, UK) for 15 min at RT. The arrays were washed with TBS-T, probed with Alexa647-Streptavidin ((Thermo Fisher Scientific, Waltham, MA, USA) diluted in TBS-T-BSA for 1 h at RT and washed again in TBS-T. For staining of total protein, arrays were incubated for 15 min in 7% acetic acid and 10% methanol, rinsed twice in water, incubated for 10 min in Sypro Ruby (Thermo Fisher Scientific, Waltham, MA, USA) and rinsed again. The processed slides were dried by centrifugation and scanned using a GenePix 4000B microarray scanner (Molecular Devices, San Jose, CA, USA). Spot intensity was determined with MicroVigene software (VigeneTech Inc., Carlisle, MA, USA). All primary antibodies used in RPPA had previously been tested by Western Blotting to assess their specificity for the protein of interest. For analysis, visual interpretation of the data was done to identify trends. Raw data were normalized using Normacurve [80], and each RPPA slide was median-centred and scaled. Loading effects were corrected individually for each array by correcting the dependency of the data in individual arrays on the median value of each sample over all 84 arrays using a linear regression.

### 4.14. Western Blots

A standard western blot protocol was used. Cells (600,000/6 well plate) were seeded and exposed to the compounds for the required time period (0, 0.5, 1, 2, 6 and 24 h) or DMSO as the negative vehicle control. Upon termination, cells were washed with TBS and placed on ice. Ice-cold RIPA cell lysis buffer (150 mM NaCl, 10 mM Tris-HCl, pH 7.4, 0.1% SDS, 0.5% sodium deoxycholate, 1mM EDTA, 1mM EGTA, protease inhibitor cocktail 2 (Sigma-Aldrich) and phosphatase cocktail (Sigma-Aldrich, St. Louis, MO, USA)) was added. Lysed cells were collected by scraping, and the supernatant collected via ultracentrifugation at 200,000 rpm at 4 °C for 30 min (Optima MAX-XP Beckman, Beckman Coulter, Brea, CA, USA). The Pierce^®^ BCA protein assay kit (Thermo Fisher Scientific Inc., Waltham, MA, USA) was used to determine the protein concentration of the samples as per manufacturer protocol. Protein concentration values were calculated using a BSA standard curve. Twenty micrograms cellular protein per sample were loaded into the wells together with tracking dye (Bromophenol blue, Sigma-Aldrich, St. Louis, MO, USA) (after denaturation at 96 °C for 5 min) and separated via electrophoresis on hand-cast 6%, 10% or 12% sodium dodecyl sulphate polyacrylamide gels (SDS-PAG) using the Mini-Protean 3 system (Bio-Rad Laboratories Inc., Hercules CA, USA). Each gel was run at 0.03 amps for 1 h in 1X migration buffer (25 mM Tris, 190 mM glycine, 0.1% SDS, pH8.3) against a relevant molecular size marker. Separated proteins were transferred to polyvinylidene difluoride membranes (Immobilon-P IPVH00010, Merck Millipore Corp., Darmstadt, Germany) activated in 100% ethanol, in a transfer buffer (25 mM Tris, 190 mM glycine, 20% ethanol, pH 8.3) using the Mini-Protean 3 system (Bio-Rad Laboratories Inc., Hercules CA, USA). Wet transfer was achieved by applying 100 V for 75 min. Membranes were rinsed in TBS (pH 7.4) and blocked with TBS-T with 5% BSA for 1 h. Membranes were incubated overnight at 4 °C with the relevant primary antibody (1:1000 antibody in BSA 5% in TBS-T) while agitated. Membranes were washed with TBS-T, followed by 1 h incubation (RT) with the relevant secondary antibody (anti-rabbit horseradish peroxidase conjugate (HRP) (Jackson ImmunoResearch, West Grove, PA, USA) at 1:10,000 or anti-mouse HRP (Sigma-Aldrich, St. Louis, MO, USA) at 1:3000 in TBS-T, 5% BSA). Membranes were washed with TBS-T, and protein detection was performed using the ECL prime (GE Healthcare, Chicago, IL, USA) chemicoluminescence kit. Chemiluminescence was detected on Amersham Hyperfilm ECL (GE Healthcare, Chicago, IL, USA) processed using an automated X-ray hyperprocessor (Amersham Biosciences, Little Chalfont, UK).

The following primary antibodies were used: Extracellular signal regulated kinase (Erk) p-Y204 raised in mouse and polyclonal anti-Erk raised in rabbit (both from Santa Cruz Biotechnology, Dallas, TX, USA); Phosphorylated c-Jun N-terminal kinase (JNK) p-Y185 raised in rabbit (Merck Millipore Corp., Darmstadt, Germany); phosphorylated mammalian target of rapamycin (mTOR) p-S2448 raised in rabbit (Santa Cruz Biotechnology, Dallas, TX, USA), c-Myc raised in mouse (Santa Cruz Biotechnology, Dallas, TX, USA) and phospho Akt S-473 raised in rabbit (Cell Signaling Technology, Danvers, MA, USA). Mouse monoclonal IgG_2α_ heat shock protein 90 (HSP90 (F-8) (Santa Cruz Biotechnology, Dallas, TX, USA) and anti-cofilin raised in rabbit (Cell Signaling Technology, Danvers, MA, USA) were used as loading control standards.

Species specific secondary antibodies labelled with horseradish peroxidase were used to obtain a signal, and densitometry analysis was done for semi-quantification using Image-J software (NIH, Bethesda, MD, USA). Blots were adjusted against loading controls and expressed as fold decrease or increase as compared to DMSO vehicle-control cells. As the expression of cofilin and HSP 90 remained relatively stable within cells exposed to the compounds under investigation, either or both of them were used as a loading control standard.

### 4.15. Statistical Analysis

Quantitative studies: RPPA and flow cytometry. Data obtained from three independent experiments are shown as the mean standard deviation (SD) and were analysed for significance using the analysis of variance (ANOVA)-single factor model followed by a two-tailed Student’s *t*-test. Means are presented in bar charts, with T-bars referring to standard deviations. *p* Values < 0.05 were regarded as statistically significant and have been indicated by an asterisk (*). For flow cytometry, data from at least 10,000 cells were analysed employing Kaluza^®^ flow analysis software version 1.3 (Beckman Coulter, Brea, CA, USA).

Qualitative studies: microscopy and Western blots. Morphological studies by means of microscopy were conducted and a minimum of 5 representative images were captured from each sample. Experiments were repeated three times, with each sample being performed minimally in duplicate. Qualitative data were obtained from microscopic techniques and semi-qualitative data were obtained from the Western blots using Image J software (NIH, Bethesda, MD, USA). Western blots were repeated at least 3 times.

## 5. Conclusions

It can be thus concluded that ESE-15-one and ESE-16 have reversible time-dependent cytotoxic effects on HeLa and MDA-MB-231 cells. These compounds induced programmed cell death involving complex cross talk between the signals resulting from abrogated microtubule dynamics, cell cycle regulation, apoptosis and autophagic pathways. Accumulation of ROS appears to link many of these pathways. ESE-15-one and ESE-16 are two 2-ME analogues, designed to increase the parent compound’s potency and oral bioavailability. The two compounds bind to the colchicine binding site of tubulin, abrogating microtubule dynamics and anatomy relatively rapidly after treatment. Subsequently, a complex network of signalling cascades induced programmed cell death in a temporal sequenced regulation. Cell cycle check point signalling indicate a pause at both the G_1_/S and G_2_/M transitions, with clear involvement of pRb and p27^Kip1^ signalling. The intrinsic apoptotic pathway is characterized by increased ROS formation, and mitochondrial outer membrane permeabilization. The hypothesis was generated that autophagy was dysregulated, possibly due to perturbations in autophagic flux consequent to abrogated microtubule dynamics, and contributed to cell death via the Akt/mTOR, Erk1/2 and p-JNK pathways which are also involved in apoptosis. Inhibition of the autophagy decreased the cytotoxic effect of the drugs, implicating its role in cell death rather than conferring a survival advantage to the drug-exposed cells. Follow-on experiments would include a time-dependent study on how 3-MA effects the induction of cell death initiated by the compound treatment, together with pan-caspase- and ROS inhibitors, which would shed more light on their exact roles in the cytotoxic effects observed. Some differences in signalling induction were seen between HeLa and MDA-MB-231 cells and between the two drugs themselves. Future research will investigate the effect of these compounds on the actin skeleton and translation of the potential anti-cancer effects into in vivo settings.

## Figures and Tables

**Table 1:** RPPA results indicating the temporal involvement of potential signalling pathways in response to ESE-15-one and ESE-16 exposure to HeLa and MDA-MB-231 cells for 24 h. Indicated is the antibody tested, the pathway involved, and the visual trend observed in expression. (↑ indicates increase; ↑↑ more pronounced increase; ↓ decrease; ↓↓ more pronounced decrease; - no change from control; ? indicates possibility/unconvincing trend).

Antibody name	Pathway(s)	HeLa		MDA-MB-231	
		ESE-15-One	ESE-16	ESE-15-One	ESE-16
Beclin-1	Autophagy, apoptosis	-	-	↑1–2 h	↑1–2 h
Puma (C-Term)	Autophagy, apoptosis	-	-	↑ 0.5–6 h	↑ 0.5–6 h
TRAIL	Apoptosis	? Mild ↑ 0.5-6 h	?	mild ↑ over 24 h	mild ↑ over 24 h
P-PAK1 (S144)	Cytoskeleton, cell cycle, apoptosis	↑ 1–6 h	-	↑ 1–6 h	-
P-Rb (S807/811)	Checkpoint, cell cycle	↑↑ over 24 h	↑↑ over 24 h	↑ over 24 h	↑ over 24 h
P-p27 KIP1 (T198)	Checkpoint, cell cycle	↑↑ over 24 h	↑↑ over 24 h	↑↑ over 24 h	↑↑ over 24 h
p15 INK4B	Cell cycle	↑ up to 2 h then plateau	↑ up to 2 h then plateau	↑ up to 2 h then plateau	↑ up to 2 h then plateau
Akt	PI3K pathway	? Mild ↑ 0.5–2 h	-	? Mild ↑ 0.5–2 h	-
P-PTEN (S380/T382/383)	PI3K pathway	? Mild ↑ 16–24 h	? Mild ↑ 16–24 h	-	-
FOXO1 (C29H4)	PI3K pathway	↓↓ over 24 h	↓↓ over 24 h	mild ↓ over 24h	mild ↓ over 24h
PI3 Kinase p110 β-subunit	PI3K pathway	-	-	↑ 1–6 h	↑1–6 h
P-Akt (S473) (193H12)	PI3K pathway	↓ over 24 h	↓ over 24 h	-	-
P-Akt substrate (RXRXX S/T)	PI3K pathway	↑ up to 2 h	↑ up to 2 h	↑ 1–2 h	↑ 1–2 h
P-mTOR (S2448)	PI3K pathway	↑ 0.5–6h, then ↓	↑ 0.5–6h, then ↓	↑ 0.5–6h, then ↓	↑ 0.5–6h, then ↓
P-p70 S6 kinase (T421/S424)	PI3K pathway	↑ 12–24 h	↑ 12–24 h	Mild ↑ 12–24 h	Mild ↑ 12–24 h
P-Stat3 (S727)	JAK/STAT signalling	-	-	Mild ↑ 0.5–6 h	Mild ↑ 0.5–6 h
P-Smad3 (S423/425)	TGF-β signalling	? ↑1–6 h	? ↑ 1–6 h	↑ 1–6 h	↑ 1–6 h
Proteins apparently not affected by compound exposure	P-p53 (S15), β-catenin (6B3), Dvl3, PKA C-α, P-AMPK β1 (S181), P-NF-kB p65 (S536)				

## Data Availability

Supporting data is provided as supplementary data.

## Supplementary Materials

The supplementary data referred to within the text are available online. Document: Mercier et al. supplementary data, Video S1: Time-lapse imaging.

## Sample Availability

Samples of the compounds are not commercially available.
