# Supplementary material for: Characterization of Signalling Pathways That Link Apoptosis and Autophagy to Cell Death Induced by Estrone Analogues Which Reversibly Depolymerize Microtubules
Source: Molecules. 2021 Jan 29;26(3):706. doi: 10.3390/molecules26030706 (PMC7866274; doi:10.3390/molecules26030706)
Supplement: Supplementary file 1 [file molecules-26-00706-s001.zip › Mercier et al. Supplementary data document_RS.pdf]

Mercier *et al*. Supplementary methods and data Sept 2020

1. Supplementary methods

S1. Signal transduction: Reverse phase protein array platform

Table S1. Antibodies used in RPPA analysis. Sp = species in which antibody was raised (R=rabbit, M=mouse); suppliers: CST= Cell Signaling Technology Corp.; Upstate (Merck Millipore Corp.); BD = BD Biosciences; Sigma = Sigma-Aldrich; Ref = reference catalogue number.

Name	Sp	Supplier	Ref	Dilution	Pathways
**Autophagy/apoptosis**					
**Tumour necrosis factor (TNF)-related apoptosis-inducing ligand (TRAIL)**	R	CST	'3219 (C92B9)'	1/1000	Apoptosis
**p53 upregulated modulator of apoptosis (PUMA) (C-Terminal)**	R	Epitomics	'1652-1'	1/5000	Autophagy, Apoptosis
**Beclin-1**	R	CST	'3738'	1/500	Autophagy, Apoptosis
**Phospho-p21-activating kinase 1 (PAK1) (serine (Ser) 144)**	R	Epitomics	'1705-1'	1/5000	Cytoskeleton, Cell cycle, Apoptosis
**DNA repair/ checkpoint control/ cell cycle**					
**Phospho-p53 (Ser 15)**	R	CST	'9284'	1/1000	DNA repair, Checkpoint, Cell cycle, Apoptosis
**Phospho-retinoblastoma (pRb) (Ser 807/811)**	R	CST	'9308'	1/1000	Checkpoint, Cell cycle
**Cyclin-dependent kinase 4 inhibitor B (p15 ^{INK4B})**	R	CST	'4822'	1/500	Cell cycle
**Phospho-cyclin-dependent kinase (Cdk) inhibitor p27 (p27^{Kip1}) (threonine (Thr) 198)**	R	Abcam	'ab64949'	1/1000	Checkpoint, Cell cycle
**Phosphoinositide 3-kinase (PI3K) pathway**					
**Protein kinase B (PKB)/Akt**	R	CST	'9272'	1/1000	PI3K pathway
**Phospho-phosphatase and tensin homolog (PTEN) (Ser380/Thr382/383)**	R	CST	'9554'	1/1000	PI3K pathway
**Phospho-Akt Substrate (RXRXX Ser/Thr)**	R	CST	'9614 (110B7e)'	1/20000	PI3K pathway
**Phospho-mammalian target of rapamycin (mTOR) (Ser2448)**	R	Abcam	'ab109268'	1/1000	PI3K pathway
**Phospho-p70 S6 kinase (Thr421/Ser424)**	R	Upstate (Millipore)	'04-393'	1/1000	PI3K pathway
**Phospho-Akt (Ser473) (193H12)**	R	CST	'4058'	1/250	PI3K pathway
**PI3 Kinase p110 subunit Beta**	R	CST	'3011'	1/1000	PI3K pathway
**Forkhead box protein O1 (FOXO1) (C29H4)**	R	CST	'2880'	1/1000	PI3K pathway
**Wnt/Notch pathway**					
**Segment polarity protein dishevelled homolog 3 (Dvl3)**	R	CST	'3218'	1/1000	Wnt/Notch pathway
**Beta catenin (6B3)**	R	CST	'9582'	1/2000	Wnt/Notch pathway
**Ca/cAMP signalling**					
**Protein kinase A (PKA)-catalytic subunit alpha**	R	Epitomics	'2113-1'	1/3000	Ca/cAMP signalling

**AMPK pathway**					
**Phospho-adenosine monophosphate (AMP)-activated protein kinase (AMPK) beta1 (Ser181)**	R	Epitomics	'2271-1'	1/1000	AMPK pathway
**Janus kinase (JAK)/signal transducer and activator of transcription (STAT) signalling**					
**Phospho- STAT3 (Ser727)**	R	CST	'9134'	1/1000	JAK/ STAT signalling
**NFκB signalling**					
**Phospho-NF-κB p65 (Ser536)**	R	CST	'3033'	1/1000	NFκB signalling
**Transforming growth factor (TGF)- β signalling**					
**Phospho- mothers against decapentaplegic homolog 3 (Smad3) (Ser423/425)**	R	Epitomics	'1880-1'	1/1000	TGF- β signalling

2. Supplementary results

S2.1. Cell cycle analysis

Flow cytometric analysis was done on the ESE-15-one and ESE-16-exposed HeLa- and MDA-MB-231 cells for 2- and 24-hours. A minimum of 10 000 cells were analysed per sample and the data plotted as FL3 Lin (X-axis) against number of cells (Y-axis) using Kaluza 1.3 software (Beckman Coulter, Ca, USA) (representative histograms displayed in figure 26.A). Data accumulated from a minimum of three biological repeats was done using the (ANOVA-single factor model and a two-tailed Student's *t*-test (statistics shown in table S2).

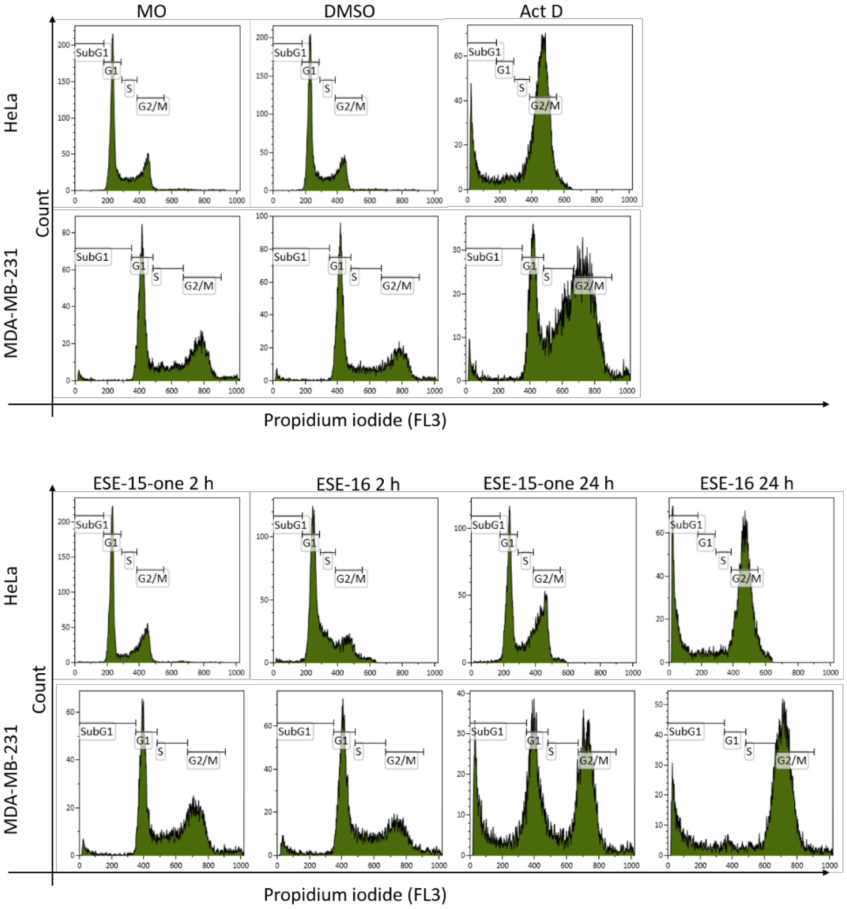

Figure S1. Representative cell cycle histograms of HeLa and MDA-MB-231 cells exposed to the compounds and controls for 2- and 24-hours. Cells propagated in medium only (MO) and exposure to the DMSO vehicle served as negative controls and did not differ in their distributions. Actinomycin D (Act D) was a positive apoptosis control. No significant changes were detected in the cell cycle distribution after a 2-hour exposure to ESE-15-one and ESE-16. At 24 hours, a significant decrease in cells in G1 was detected, together with an increase in G2/M and sub-G1 indicating a metaphase block and apoptosis respectively.

Table S2. Cell cycle analysis of HeLa and MDA-MB-231 cells. Statistical data were generated using Kaluza 1.3 software (Beckman Coulter, Ca, USA) and analysed using the ANOVA-single factor model and a two-tailed Student's t-test. Displayed are the average percentages of cells (three biological repeats) in each phase of the cell cycle, the standard deviation (Std dev) and the P value calculated to determine statistical significance when compared to the DMSO vehicle control. (MO = cells propagated in growth-medium only; Act D = actinomycin D)

Agent of exposure		MO	DMSO	Act D	ESE-15-one 2 h	ESE-16 2 h	ESE-15-one 24 h	ESE-16 24 h
**Cell cycle stages**		**HeLa cell line**						
Sub-G_1	Average	0.33	1.19	18.32	5.42	5.66	18.99	22.80
	Std dev	0.15	1.15	4.53	0.82	1.90	1.70	3.95
	P value	3.16E-01		4.97E-03	1.38E-01	1.52E-01	3.05E-03	1.36E-03
G_1	Average	58.34	62.14	42.50	61.57	63.02	40.43	7.04
	Std dev	3.43	3.82	3.54	5.05	8.51	3.32	3.08
	P value	1.90E-01		3.79E-03	8.83E-01	8.59E-01	5.45E-04	5.12E-07
S	Average	13.45	13.21	10.10	9.89	12.56	14.43	7.54
	Std dev	1.89	2.52	1.85	1.25	2.04	2.67	2.42
	P value	8.81E-01		1.34E-01	1.66E-01	7.72E-01	5.30E-01	1.75E-02
G_2/M	Average	23.44	20.16	29.00	21.17	17.31	35.72	49.12
	Std dev	4.33	4.07	1.41	2.59	2.40	6.46	5.79
	P value	3.13E-01		4.69E-02	7.72E-01	4.26E-01	6.54E-03	5.41E-04
		**MDA-MB-231 cell line**						
Sub-G_1	Average	2.93	3.60	13.88	3.65	7.23	28.52	23.61
	Std dev	1.62	2.46	0.62	0.12	0.95	5.55	0.06
	P value	7.12E-01		2.16E-03	9.83E-01	1.53E-01	5.48E-03	1.64E-03
G_1	Average	48.86	51.50	26.16	47.82	52.07	26.21	6.51
	Stddev	1.91	0.92	0.62	3.34	2.62	3.09	0.62
	P value	9.71E-02		5.93E-05	1.46E-01	7.40E-01	7.39E-04	1.06E-05
S	Average	14.19	14.14	22.94	16.43	14.51	8.05	8.51
	Stddev	0.81	0.98	2.16	2.57	1.91	0.64	1.36
	P value	9.49E-01		3.01E-03	2.34E-01	7.78E-01	8.37E-04	1.18E-02
G_2/M	Average	33.79	30.60	36.80	29.36	27.91	41.67	59.18
	Stddev	2.12	2.65	1.51	3.95	1.64	5.27	3.83
	P value	1.80E-01		2.46E-02	6.74E-01	2.09E-01	3.15E-02	2.06E-03

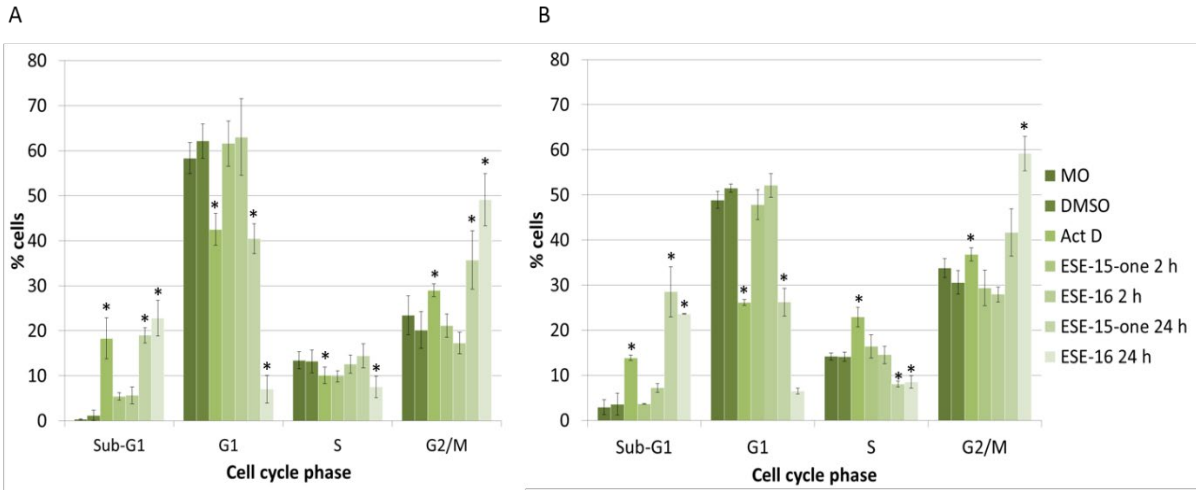

Figure S2. Bar graphs representing the stages of the cell cycle after a 2- and 24-hour exposure of HeLa and MDA-MB-231 cells to ESE-15-one and ESE-16. A. HeLa- and B. MDA-MB-231 cell cycle analysis. Actinomycin D (Act D) served as a positive apoptosis control. No statistical changes were evident in the cell cycle distributions after a 2-hour drug-exposure in both cell lines. A 24-hour drug-exposure resulted in a significant decrease in cells in G_1 with a concomitant increase in G_2/M and sub-G_1 for both compounds and cell lines. Bars represent the mean % cells for 3 biological repeats, standard deviation indicated by error bars, and **p* < 0.05 as compared to the DMSO-exposed samples. (MO = cells propagated in medium only)

S2.2. Apoptosis induction: Annexin V quantification by flow cytometry

In order to detect induction of apoptosis in HeLa and MDA-MB-231 cells exposed to ESE-15-one and ESE-16 for 2- and 24-hours, flow cytometric detection of Annexin V was done (representative dot-plots shown in Figure S4). A 2-hour exposure to both drugs neither had a statistically significant effect on viability nor apoptosis induction in both cell lines. A 24-hour exposure to the analogues resulted in statistically significant decreased cell viability with a concurrent increase in apoptosis induction in both cell lines (statistics summarised in table S3; bar graph in figure S5). HeLa cells exposed to ESE-15-one and ESE-16 resulted in a drop in viability to 64.0 ± 8.6% and 52.0 ± 0.4% respectively. The same drug-exposure resulted in a drop in viability to 52.4 ± 7.9% and 53.8 ± 2.9% in MDA-MB-231 cells. Apoptosis induction increased to 32.5 ± 5.8% and 42.8 ± 4.4% after ESE-15-one exposure in HeLa and MDA-MB-231 cells respectively. ESE-16 exposure increased apoptosis induction to 31.2 ± 4.4% and 43.3 ± 5.8% in HeLa and MDA-MB-231 cells. None of the treatment conditions demonstrated a significant change in necrosis.

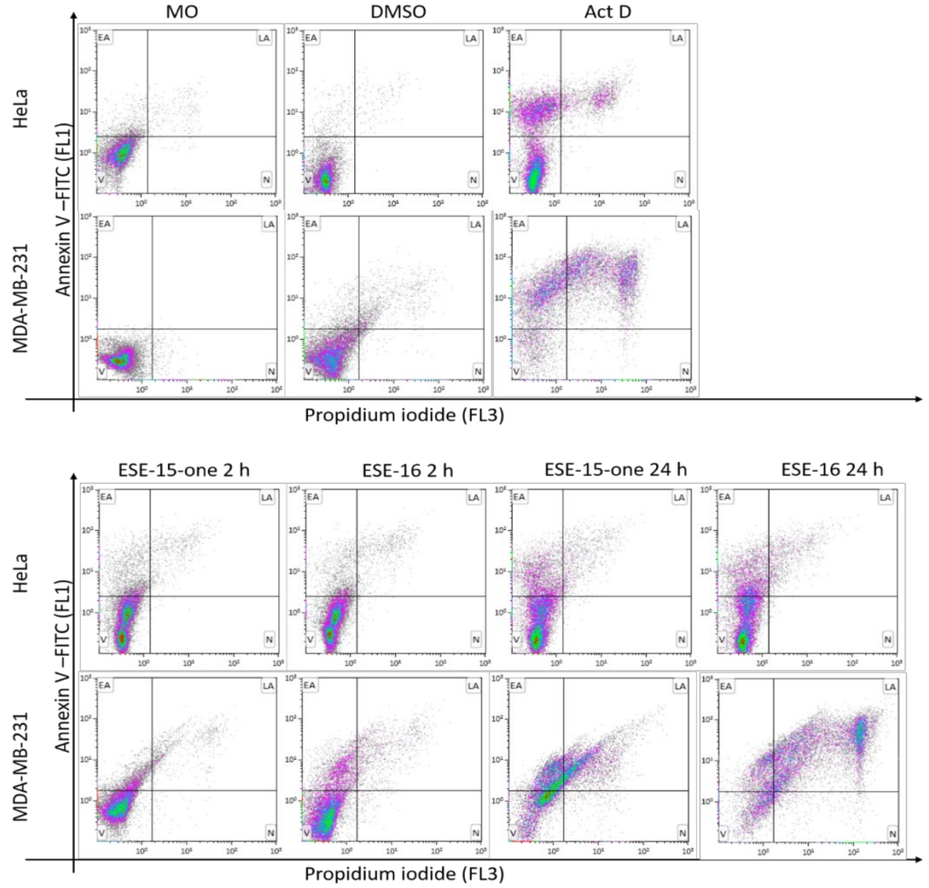

Figure S3. Representative flow cytometric dot-plots of Annexin V-FITC in HeLa and MDA-MB-231 cells exposed to the compounds for 2- and 24-hours. Cells propagated in medium only (MO) and exposed to DMSO served as negative controls. Actinomycin D (Act D) was a positive apoptosis control. (V = viable cells; EA = early apoptosis; LA = late apoptosis; N = necrosis)

Table S3. Annexin-V analysis of drug-exposed HeLa and MDA-MB-231 cells. Data were generated using Kaluza 1.3 software (Beckman Coulter) and analysed using the ANOVA-single factor model and a two-tailed Student's t-test. Displayed are the average percentages of cells (three biological repeats) which are viable, undergoing apoptosis or necrotic. (MO = cells propagated in growth-medium only; Act D = actinomycin D)

Agent of exposure		MO	DMSO	Act D	ESE-15-one 2 h	ESE-16 2 h	ESE-15-one 24 h	ESE-16 24 h
% cells		HeLa						
Viable	Mean	84.39	87.10	64.01	90.52	86.95	64.03	52.03
	Std dev	0.21	4.67	4.24	3.56	6.35	8.59	0.38
	P value		6.91E-01	2.10E-02	1.35E-01	6.25E-01	9.42E-03	8.97E-05
Apoptosis	Mean	13.75	11.15	37.78	7.94	11.46	32.49	31.24
	Std dev	1.67	4.66	2.12	1.51	6.50	5.82	4.38
	P value		5.21E-01	6.25E-03	6.73E-02	6.73E-01	2.41E-02	3.41E-02
Necrosis	Mean	1.87	1.60	2.21	1.16	0.93	1.47	1.65
	Std dev	1.46	1.09	0.71	0.97	0.70	1.15	0.82
	P value		8.30E-01	7.92E-01	5.52E-01	3.89E-01	7.55E-01	7.15E-01
		MDA-MB-231						
Viable	Mean	81.12	79.10	39.61	79.90	78.86	52.41	53.80
	Std dev	4.41	2.18	6.25	3.16	3.52	7.91	2.88
	P value		6.20E-01	4.11E-03	7.38E-01	5.66E-01	4.63E-02	1.81E-02
Apoptosis	Mean	11.35	18.76	53.47	19.55	26.18	42.79	43.26
	Std dev	5.07	4.91	10.73	3.36	1.61	4.38	5.81
	P value		2.76E-01	3.75E-02	1.11E-01	5.87E-02	2.19E-02	2.80E-02
Necrosis	Mean	1.26	1.65	1.85	0.55	1.10	0.70	2.28
	Std dev	0.84	0.93	1.10	0.25	0.72	0.18	1.20
	P value		8.03E-01	5.93E-01	4.33E-01	8.90E-01	6.30E-01	5.54E-01

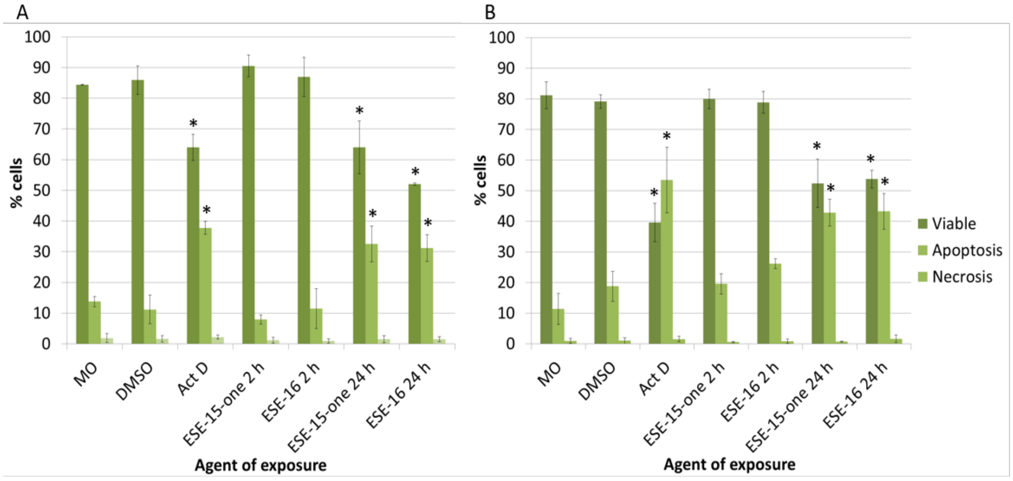

Figure S4. Bar graphs representing cell viability and apoptosis induction after a 2- and 24-hour exposure of HeLa (A) and MDA-MB-231 (B) cells to ESE-15-one and ESE-16. No statistical changes were evident in the cell viability after a 2-hour exposure in both cell lines. A 24-hour drug-exposure resulted in a significant decrease in viable cells with a concomitant increase in cells undergoing apoptosis from exposure to both compounds in HeLa and MDA-MB-231 cells. Bar charts represent the mean % cells for 3 biological repeats, SD indicated by error bars; *$p < 0.05$. (MO = cells propagated in medium only)

S2.3. Autophagic vacuole quantification via autophagy-related light chain 3 (LC3B) protein quantification

Tamoxifen was used as a positive autophagy control. No differences were detected between the vehicle-exposed cells and cells exposed to growth medium only, nor cells exposed to the compounds for 2 h in both cell lines. ESE-15-one-exposure for 24 hours increased LC3B detection significantly by 1.59 ± 0.29-fold in HeLa cells and by 2.61 ± 0.46-fold in MDA-MB-231 cells. Similarly, ESE-16-exposure increased LC3B detection by 1.63 ± 0.15-fold in HeLa cells and 2.16 ± 0.43-fold in MDA-MB-231 cells after 24 hours.

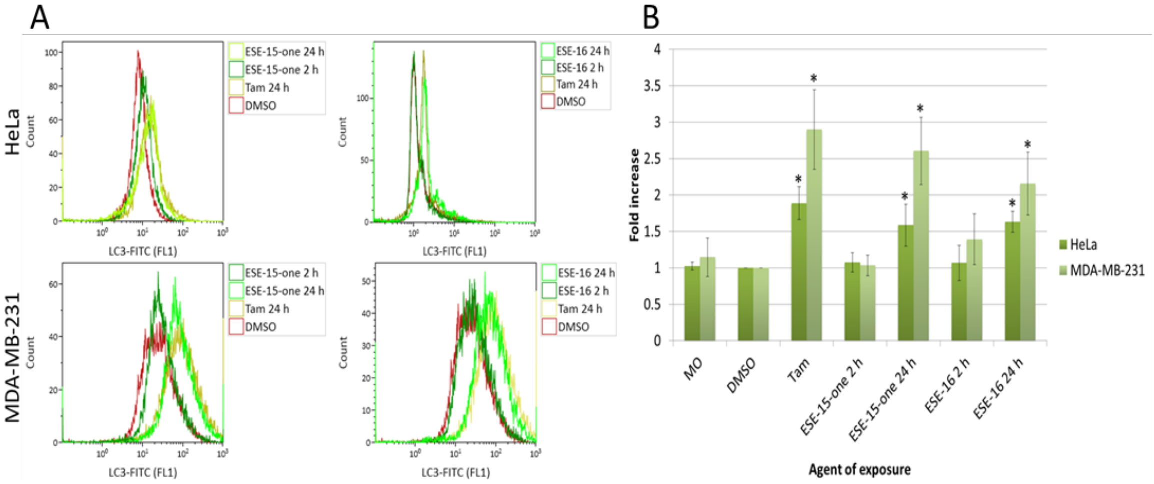

Figure S5. Overlay histograms (A) and bar chart (B) demonstrating the fold-increase in LC3-detection in HeLa and MDA-MB-231 cells exposed to the novel compounds. An increase in LC3B expression was observed at the 24-hour exposure. Bar charts represent the mean fold-increase over 3 biological repeats, standard deviation indicated by error bars, and *$p < 0.05$.

S2.4. Attenuation of autophagy decreases the cytotoxic effects of ESE-15-one and ESE-16 in MDA-MB-231 cells

In order to quantify the effect of autophagy inhibition on the cytotoxicity of ESE-15-one and ESE-16, flow cytometric quantification of Annexin V was done. MDA-MB-231 cells were exposed to ESE-15-one and ESE-16 for 2- and 24 h. The effect of autophagy inhibition on MDA-MB-231 cells during ESE-15-one and ESE-16-exposure was similar to that in HeLa cells. ESE-15-one exposure resulted in a decreased cell viability (52.41 ± 7.91%) and increase in apoptosis (42.79 ± 4.38%), a response which was attenuated by the addition of 3MA resulting in 86.45 ± 0.64% viable- and 12.38 ± 0.45% apoptotic cells, values which were not significantly different from the DMSO negative control. Wortmannin addition resulted in a partial reduction in this response, with 64.88 ± 2.96% viable- and 32.46 ± 2.36% apoptotic cells. Similarly, ESE-16-exposed cells demonstrated relative resistance to the compound when 3MA was added, significantly increasing the viability (73.92 ± 4.23%) and decreasing the apoptotic fraction (24.03 ± 2.64%) when compare to the drug-only-exposed cells (53.80 ± 2.88% viable and 43.26 ± 5.81% apoptotic). Wortmannin similarly increased the viability to 74.88 ± 0.37% and significantly decreased the number of cells undergoing apoptosis (23.13 ± 2.52%).

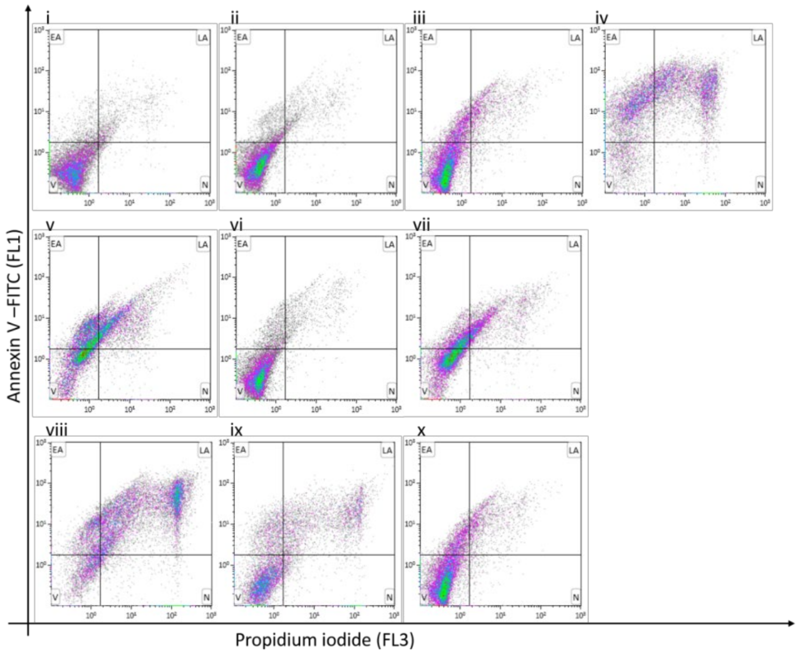

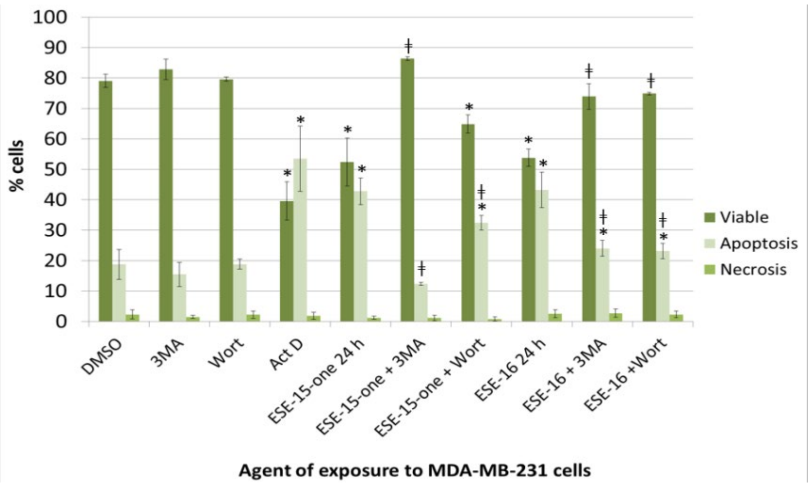

Figure S6. Representative Annexin V scatter plots and bar chart of MDA-MB-231 cells exposed to the compounds in the presence or absence of autophagy inhibition. Cells exposed to DMSO (i), 3MA (ii) and wortmannin (iii) showed no effect on cell viability. Actinomycin D (iv) was the positive apoptosis control. ESE-15-one exposure for 24 h (v) showed decreased cell viability with a concomitant increase in apoptotic cells. This response was reduced in the presence of autophagy inhibition with 3MA (vi) and wortmannin (vii). ESE-16-exposure (viii) demonstrated a similar response, but less so in the presence of 3MA (ix) and wortmannin (x). (V = viable cells; EA = early apoptosis; LA = late apoptosis; N = necrosis). SD indicated by error bars; *$p < 0.05$ when compared to the DMSO vehicle control; ‡$p < 0.05$ when autophagy-inhibited samples compared to the relevant drug-exposed samples.

Table S4. LC3B quantification in drug-exposed HeLa and MDA-MB-231 cells with- and without the inhibition of autophagy. Statistical data were generated using Kaluza 1.3 software (Beckman Coulter, Ca, USA) and analysed using the ANOVA-single factor model and a two-tailed Student's *t*-test. The average fold-increase (three biological repeats) which was detected in drug-exposed cells either alone, or in the presence of 3MA or wortmannin, is shown. The standard deviation (Std dev) and the *p* values were calculated to determine statistical significance when compared to the DMSO vehicle control (*) or when the autophagy-inhibited samples were compared to the relevant drug-exposed samples (ǂ).

**Agent of exposure**	**DMSO**	**3MA**	**Wort**	**Tam**	**Tam + 3MA**	**Tam + Wort**	**ESE-15-one 24 h**	**ESE-15-one + 3MA**	**ESE-15-one + Wort**	**ESE-16 24 h**	**ESE-16 + 3MA**	**ESE-16 + Wort**
**HeLa cells**												
**Average**	1	1.00	0.93	1.89	0.99	0.89	1.59	1.17	1.30	1.63	1.26	1.34
**Std dev**	0	0.09	0.01	0.22	0.20	0.09	0.29	0.14	0.07	0.15	0.09	0.15
***P* value (*) cf DMSO**		9.33E-01	1.22E-06	1.13E-03	9.63E-01	1.04E-01	1.85E-02	1.08E-01	4.74E-03	7.22E-04	4.00E-03	1.74E-02
***P* value (ǂ) cf drug**					1.03E-03	4.44E-03		3.98E-02	2.61E-01		4.47E-03	4.51E-02
**MDA-MB-231 cells**												
**Average**	1	1.14	1.07	2.90	1.68	1.97	2.61	1.14	1.60	2.16	1.23	0.97
**Std dev**	0	0.19	0.04	0.54	0.38	0.46	0.46	0.13	0.30	0.43	0.21	0.23
***P* value (*) cf DMSO**		2.92E-01	5.66E-02	3.80E-03	3.62E-02	2.84E-02	3.86E-03	1.37E-01	2.73E-02	9.62E-03	1.32E-01	8.56E-01
***P* value (ǂ) cf drug**					3.33E-02	1.45E-01		2.49E-02	3.42E-02		2.82E-02	1.38E-02

S2.5. Reverse phase protein array graphs

Individual results of the RPPA experiments in which ESE-15-one and ESE-16 (0.186 µM and 0.5 µM respectively) were used to treat HeLa and MDA-MB-231 cells in a time series.

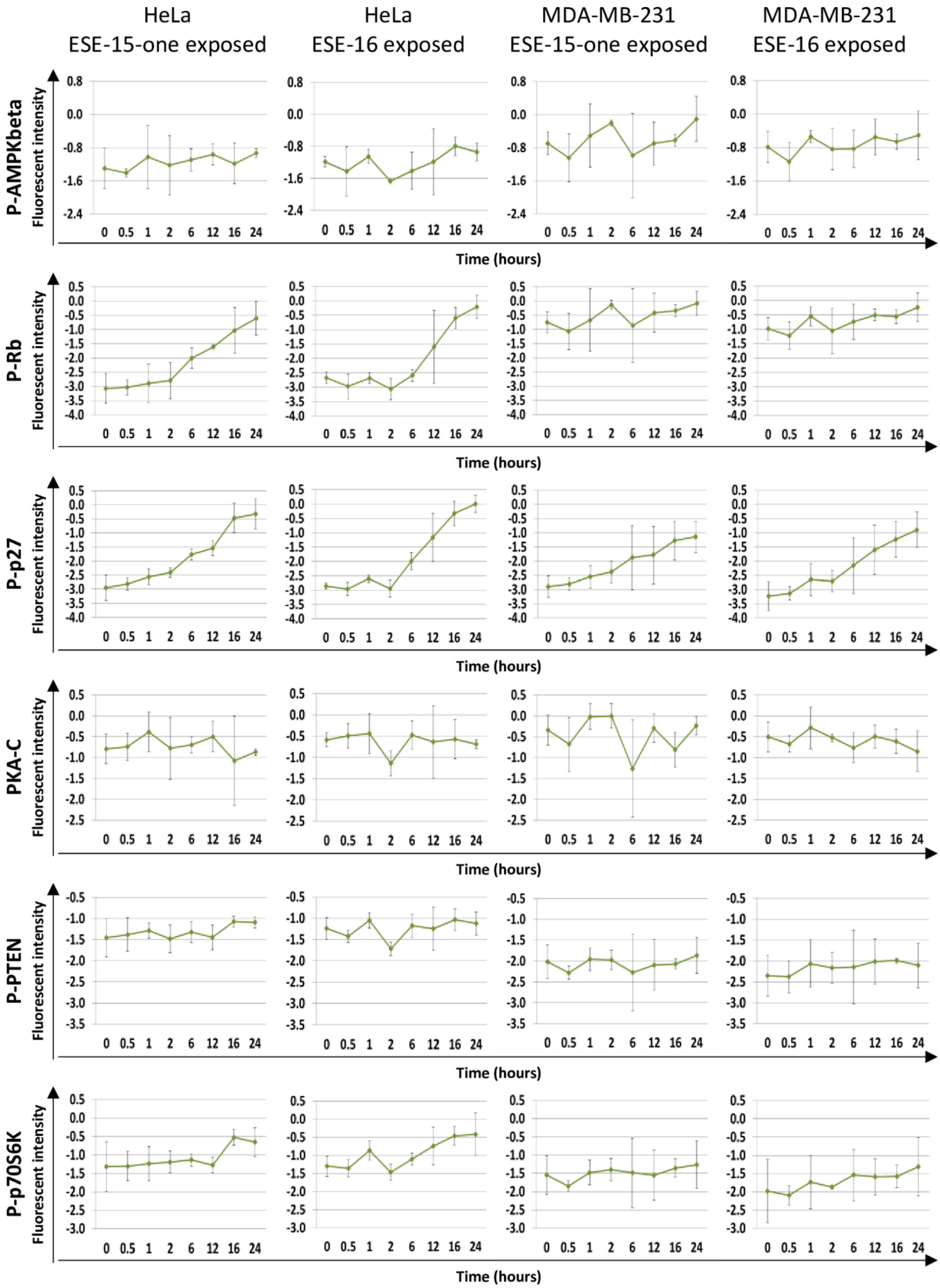
HeLa ESE-15-one exposed
HeLa ESE-16 exposed
MDA-MB-231 ESE-15-one exposed
MDA-MB-231 ESE-16 exposed
P-AMPKbeta
P-Rb
P-p27
PKA-C
P-PTEN
P-p70S6K
Fluorescent intensity
Time (hours)

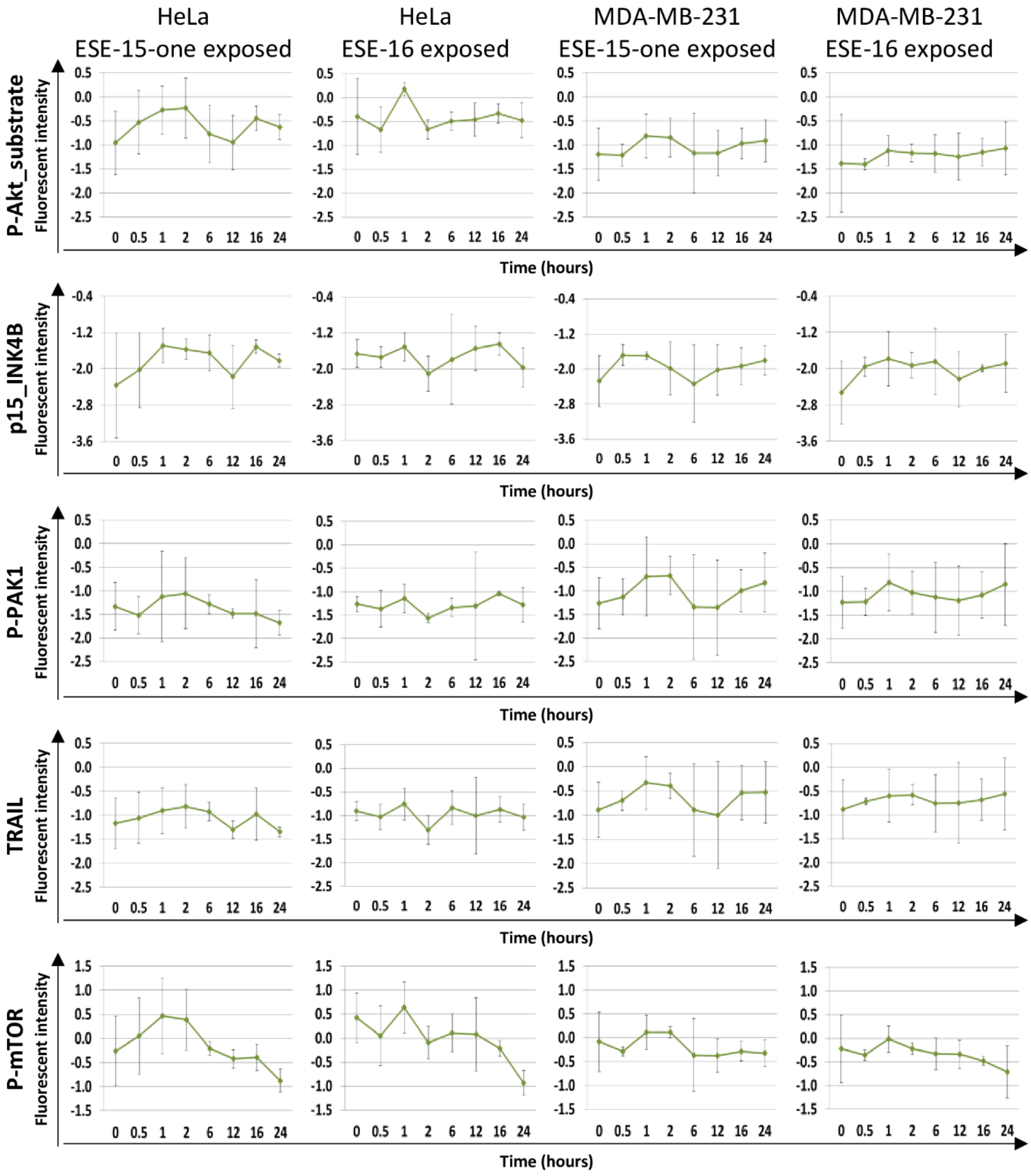
HeLa ESE-15-one exposed
HeLa ESE-16 exposed
MDA-MB-231 ESE-15-one exposed
MDA-MB-231 ESE-16 exposed
P-Akt_substrate
p15_INK4B
P-PAK1
TRAIL
P-mTOR
Fluorescent intensity
Time (hours)
0 0.5 1 2 6 12 16 24

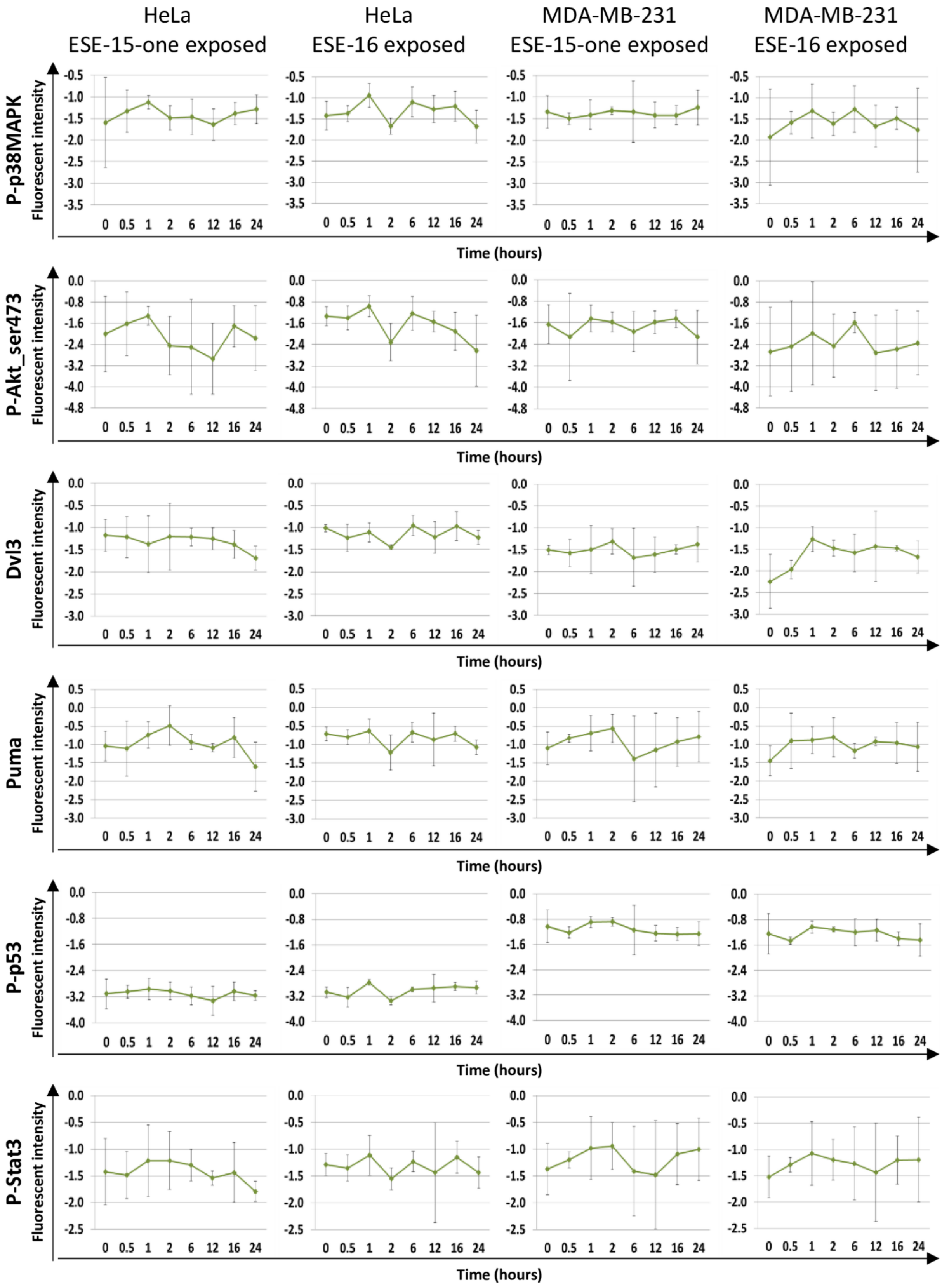

HeLa ESE-15-one exposed
HeLa ESE-16 exposed
MDA-MB-231 ESE-15-one exposed
MDA-MB-231 ESE-16 exposed
P-p38MAPK
P-Akt_ser473
Dvl3
Puma
P-p53
P-Stat3
Fluorescent intensity
Time (hours)
0 0.5 1 2 6 12 16 24

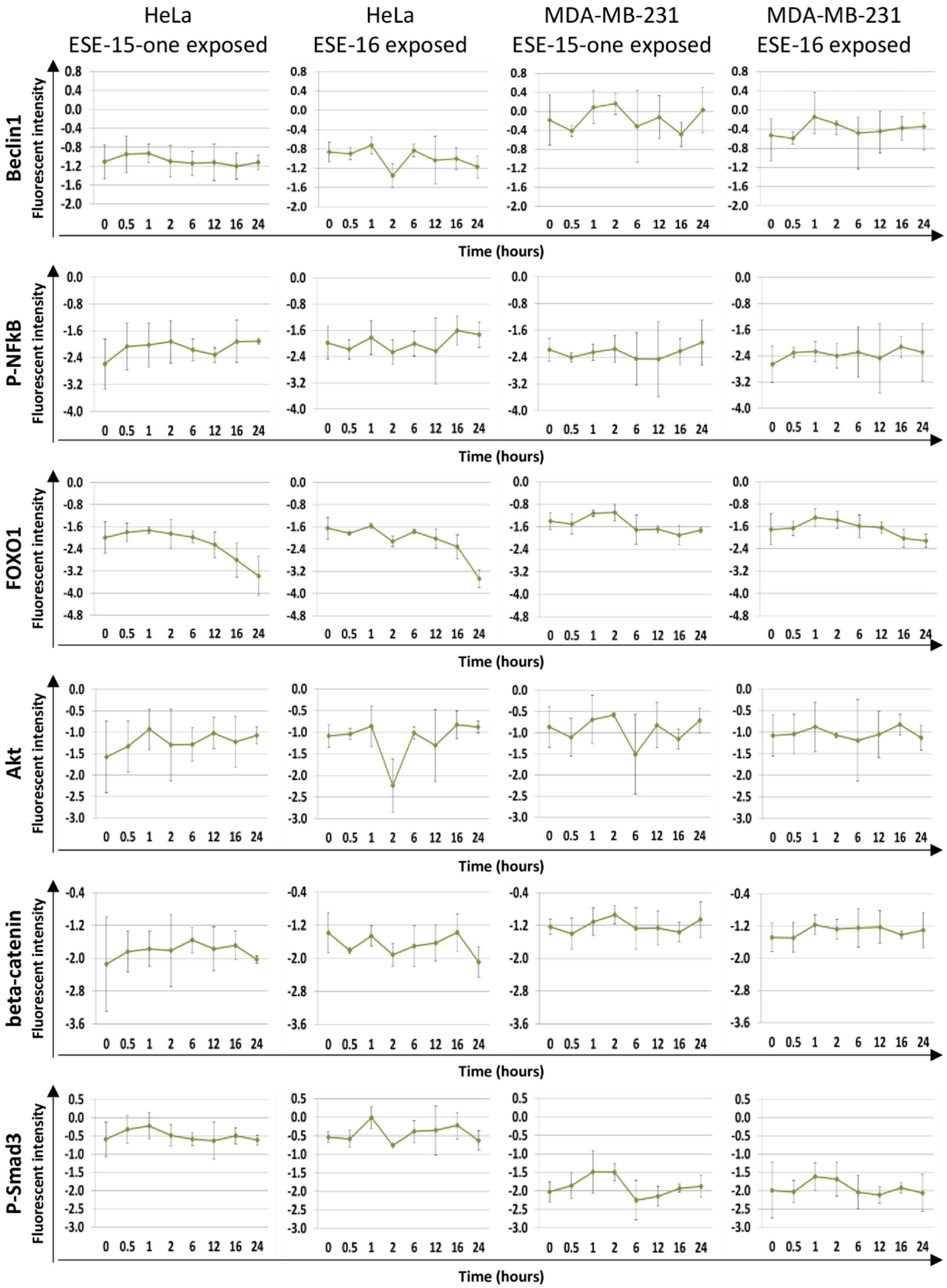

HeLa
ESE-15-one exposed
HeLa
ESE-16 exposed
MDA-MB-231
ESE-15-one exposed
MDA-MB-231
ESE-16 exposed
Beclin1
P-NFkB
FOXO1
Akt
beta-catenin
P-Smad3
Fluorescent intensity
Time (hours)
0 0.5 1 2 6 12 16 24

S2.6. Western blots

Tables of statistical analysis provided for the Western blot experiments.

Table S5. Src expression (A) and sequential Src phosphorylation (B) in HeLa and MDA-MB-231 cells exposed to the novel compounds.

A

HeLa	Fold-change	DMSO	0.5 h	1 h	2 h	6 h	24 h
ESE-15-one	Mean	1	0.82	0.81	1.01	0.86	0.62
	SEM		0.141	0.137	0.038	0.117	0.262
	P value		2.08E-01	3.28E-01	3.59E-01	5.73E-01	1.27E-02
ESE-16	Mean	1	0.80	0.67	0.65	0.72	0.33
	SEM		0.189	0.058	0.080	0.169	0.119
	P value		2.00E-01	3.57E-04	1.04E-03	6.52E-02	3.74E-04

A

MDA-MD-231	Fold-change	DMSO	0.5 h	1 h	2 h	6 h	24 h
ESE-15-one	Mean	1	0.99	1.13	0.91	0.85	0.58
	SEM		0.057	0.073	0.074	0.176	0.089
	P value		9.01E-01	8.63E-02	1.95E-01	3.53E-01	2.44E-03
ESE-16	Mean	1	0.91	1.13	0.82	0.82	0.45
	SEM		0.042	0.193	0.086	0.117	0.025
	P value		6.93E-02	4.61E-01	7.70E-02	1.26E-01	6.08E-07

B

HeLa	Fold change	DMSO	0.5 h	1 h	2 h	6 h	24 h
ESE-15-one	Mean	1	0.91	1.14	0.89	0.93	0.40
	SEM		0.172	0.102	0.064	0.056	0.188
	P value		6.46E-01	2.40E-01	1.74E-01	2.99E-01	3.39E-02
ESE-16	Mean	1	0.98	1.17	0.97	0.91	0.44
	SEM		0.164	0.084	0.216	0.208	0.113
	P value		9.22E-01	1.15E-01	8.84E-01	6.96E-01	7.50E-03

B

MDA-MD-231	Fold change	DMSO	0.5 h	1 h	2 h	6 h	24 h
ESE-15-one	Mean	1	2.61	2.54	1.38	2.17	1.85
	SEM		0.593	0.198	0.179	0.289	0.574
	P value		2.31E-02	2.48E-04	7.59E-02	6.80E-03	1.88E-01
ESE-16	Mean	1	1.14	2.98	1.44	0.77	0.58
	SEM		0.133	0.945	0.418	0.247	0.211
	P value		1.49E-01	2.67E-02	2.62E-01	2.09E-01	9.60E-02

Table S6. Erk expression (A) and sequential Erk phosphorylation (B) in HeLa and MDA-MB-231 cells exposed to the novel compounds.

A

HeLa	Fold-change	DMSO	0.5 h	1 h	2 h	6 h	24 h
ESE-15-one	Mean	1	0.96	1.02	1.00	0.95	0.82
	SEM		0.078	0.136	0.119	0.022	0.024
	P value		6.29E-01	8.42E-01	9.83E-01	8.03E-02	3.07E-04
ESE-16	Mean	1	0.95	0.99	1.00	0.97	0.81
	SEM		0.093	0.041	0.080	0.193	0.040
	P value		5.86E-01	8.65E-01	9.62E-01	8.73E-01	2.66E-03

A

MDA-MD-231	Fold-change	DMSO	0.5 h	1 h	2 h	6 h	24 h
ESE-15-one	Mean	1	0.98	1.12	0.95	1.01	0.92
	SEM		0.046	0.049	0.134	0.145	0.166
	P value		6.44E-01	2.75E-02	6.21E-01	9.21E-01	5.40E-01
ESE-16	Mean	1	0.96	0.96	1.12	1.14	0.80
	SEM		0.064	0.112	0.062	0.106	0.179
	P value		5.79E-01	6.55E-01	3.84E-02	1.29E-01	2.47E-01

B

HeLa	Fold-change	DMSO	0.5 h	1 h	2 h	6 h	24 h
ESE-15-one	Mean	1	1.24	1.61	1.11	1.25	0.46
	SEM		0.112	0.098	0.031	0.128	0.169
	P value		7.51E-02	8.31E-04	1.47E-02	3.41E-02	1.86E-02
ESE-16	Mean	1	1.55	1.63	1.66	1.29	0.38
	SEM		0.199	0.125	0.274	0.172	0.131
	P value		2.20E-02	2.32E-03	3.48E-02	9.70E-02	3.23E-03

B

MDA-MD-231	Fold-change	DMSO	0.5 h	1 h	2 h	6 h	24 h
ESE-15-one	Mean	1	0.94	2.07	3.24	1.20	1.16
	SEM		0.173	0.357	0.050	0.119	0.040
	P value		6.90E-01	4.67E-03	9.80E-11	3.95E-02	5.78E-04
ESE-16	Mean	1	1.24	2.03	1.35	0.83	0.32
	SEM		0.117	0.492	0.204	0.180	0.052
	P value		4.63E-02	1.54E-02	3.26E-02	2.11E-01	5.76E-08

Table S7. Sequential JNK phosphorylation in HeLa and MDA-MB-231 cells exposed to the novel compounds.

HeLa	Fold-change	DMSO	0.5 h	1 h	2 h	6 h	24 h
ESE-15-one	Mean	1	0.18	0.58	0.69	0.95	24.54
	SEM		0.146	0.289	0.382	0.474	8.975
	P value		2.21E-04	7.44E-03	4.67E-01	9.18E-01	3.89E-02
ESE-16	Mean	1	0.51	0.88	0.99	4.74	26.33
	SEM		0.187	0.019	0.521	0.234	5.404
	P value		3.92E-02	3.70E-03	9.80E-01	2.21E-04	4.12E-02

MDA-MD-231	Fold-change	DMSO	0.5 h	1 h	2 h	6 h	24 h
ESE-15-one	Mean	1	0.63	0.88	0.53	7.36	8.93
	SEM		0.108	0.063	0.227	0.935	1.633
	P value		1.93E-02	1.38E-01	6.90E-02	2.78E-03	7.35E-03
ESE-16	Mean	1	0.96	1.15	0.92	3.63	9.04
	SEM		0.056	0.245	0.200	1.152	0.088
	P value		5.04E-01	4.66E-01	6.47E-01	5.47E-02	3.92E-06

Table S8. Expression of c-Myc in HeLa and MDA-MB-231 cells exposed to the novel compounds.

HeLa	Fold-change	DMSO	0.5 h	1 h	2 h	6 h	24 h
ESE-15-one	Mean	1	0.81	0.83	1.17	1.20	0.77
	SEM		0.214	0.174	0.152	0.260	0.303
	P value		3.28E-01	1.79E-01	1.40E-01	2.80E-01	4.79E-01
ESE-16	Mean	1	1.04	1.07	0.97	0.70	1.01
	SEM		0.262	0.215	0.257	0.348	0.324
	P value		8.91E-01	7.02E-01	9.14E-01	3.37E-01	9.80E-01

MDA-MD-231	Fold-change	DMSO	0.5 h	1 h	2 h	6 h	24 h
ESE-15-one	Mean	1	0.96	1.17	0.89	0.69	0.66
	SEM		0.078	0.167	0.187	0.067	0.037
	P value		4.20E-01	2.69E-01	5.21E-01	2.69E-03	1.02E-04
ESE-16	Mean	1	1.00	0.99	0.98	0.98	0.52
	SEM		0.118	0.142	0.103	0.142	0.087
	P value		9.82E-01	9.64E-01	8.53E-01	8.62E-01	1.20E-03

Table S9. Sequential Akt phosphorylation in HeLa and MDA-MB-231 cells exposed to the novel compounds.

HeLa	Fold-change	DMSO	0.5 h	1 h	2 h	6 h	24 h
ESE-15-one	Mean	1	0.82	0.81	1.01	0.86	0.62
	SEM		0.141	0.137	0.038	0.117	0.262
	P value		1.24E-01	1.61E-01	8.63E-01	1.45E-01	4.23E-02
ESE-16	Mean	1	0.80	0.67	0.65	0.72	0.33
	SEM		0.189	0.058	0.080	0.169	0.119
	P value		2.00E-01	3.57E-04	1.04E-03	6.52E-02	3.74E-04

MDA-MD-231	Fold-change	DMSO	0.5 h	1 h	2 h	6 h	24 h
ESE-15-one	Mean	1	1.16	0.94	0.47	0.32	0.40
	SEM		0.017	0.121	0.263	0.114	0.198
	P value		1.04E-03	6.48E-01	1.13E-01	3.95E-03	3.96E-02
ESE-16	Mean	1	1.49	0.81	0.44	0.71	0.76
	SEM		0.029	0.042	0.181	0.177	0.069
	P value		1.97E-04	9.16E-03	3.61E-02	1.15E-01	1.80E-02

Table S10. Sequential mTOR phosphorylation in HeLa and MDA-MB-231 cells exposed to the novel compounds.

HeLa	Fold-change	DMSO	0.5 h	1 h	2 h	6 h	24 h
ESE-15-one	Mean	1	1.03	1.25	1.25	1.05	0.42
	SEM		0.220	0.177	0.098	0.138	0.186
	P value		8.72E-01	1.47E-01	2.95E-02	7.05E-01	2.11E-02
ESE-16	Mean	1	1.08	1.56	0.91	0.96	0.50
	SEM		0.026	0.222	0.083	0.143	0.166
	P value		1.68E-02	4.47E-02	3.94E-01	7.77E-01	2.44E-02

MDA-MD-231	Fold-change	DMSO	0.5 h	1 h	2 h	6 h	24 h
ESE-15-one	Mean	1	1.76	2.40	1.25	0.90	0.54
	SEM		0.038	0.142	0.134	0.099	0.086
	P value		8.45E-08	1.00E-05	4.32E-02	3.46E-01	3.40E-04
ESE-16	Mean	1	1.32	1.43	1.52	0.87	0.56
	SEM		0.081	0.064	0.235	0.259	0.168
	P value		2.90E-03	9.49E-05	2.35E-02	5.98E-01	1.17E-02

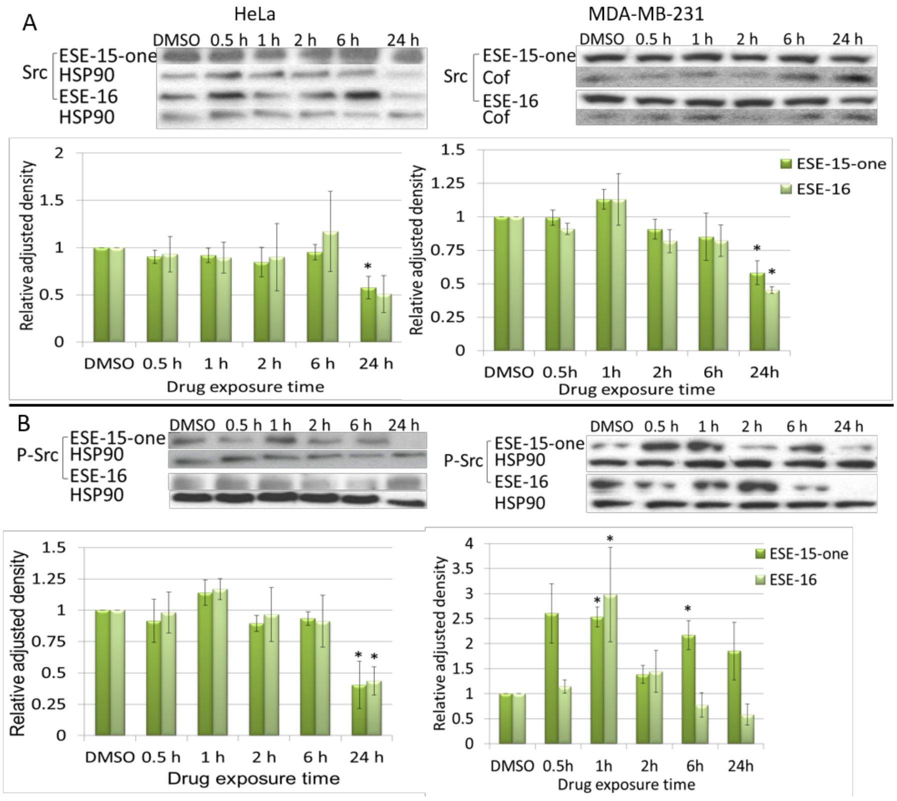

Figure S7. Src expression (A) and sequential Src phosphorylation (B) in HeLa and MDA-MB-231 cells exposed to the novel compounds. Bars represent a mean fold-increase of at least 3 repeats, the error bar show SEM, and * $p < 0.05$.

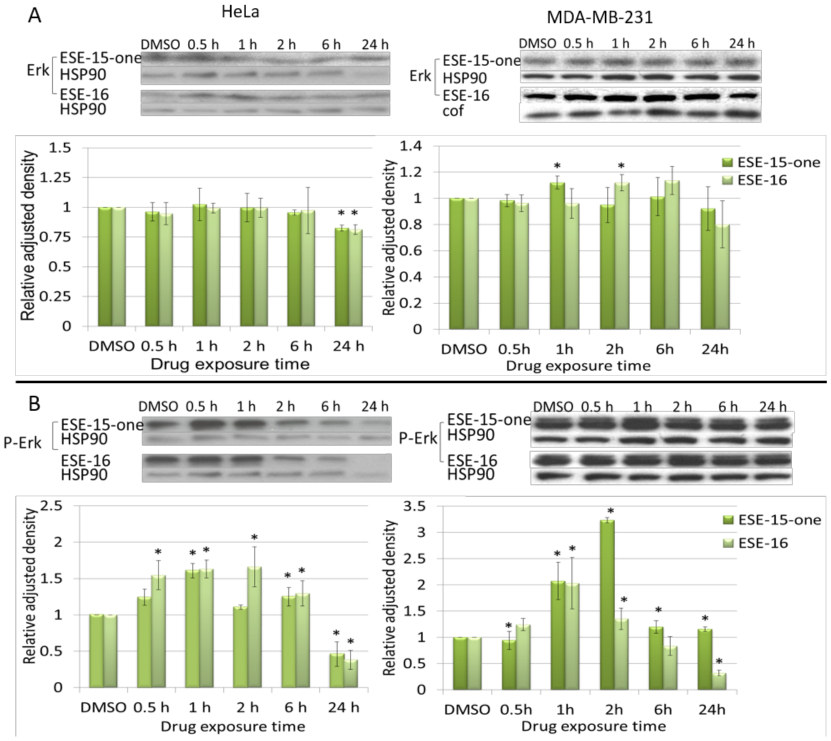

Figure S8. Erk expression (A) and sequential Erk phosphorylation (B) in HeLa and MDA-MB-231 cells exposed to the novel compounds. Bars represent a mean fold-increase of at least 3 repeats, the error bar show SEM, and *$p < 0.05$.

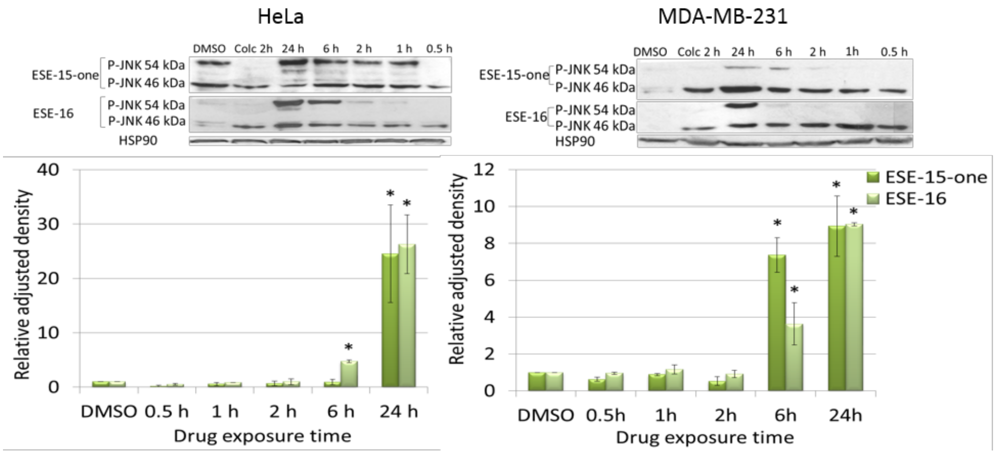

Figure S9. Sequential JNK phosphorylation in HeLa and MDA-MB-231 cells exposed to the Bars represent a mean fold-increase of at least 3 repeats of the 54 kDa isoform, the error bar show SEM, and *$p < 0.05$.

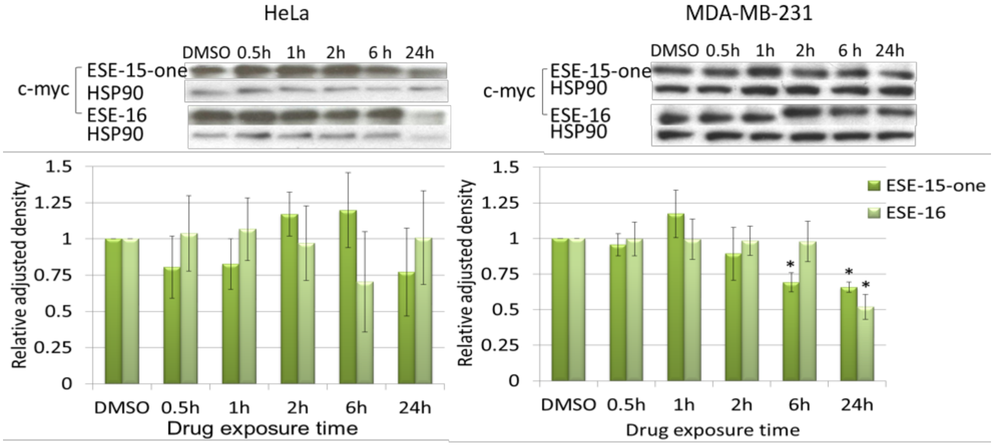

Figure S10. Expression of c-Myc in HeLa and MDA-MB-231 cells exposed to the 2-ME analogous. Bars represent a mean fold-increase of at least 3 repeats, the error bar show SEM, and *$p < 0.05$.

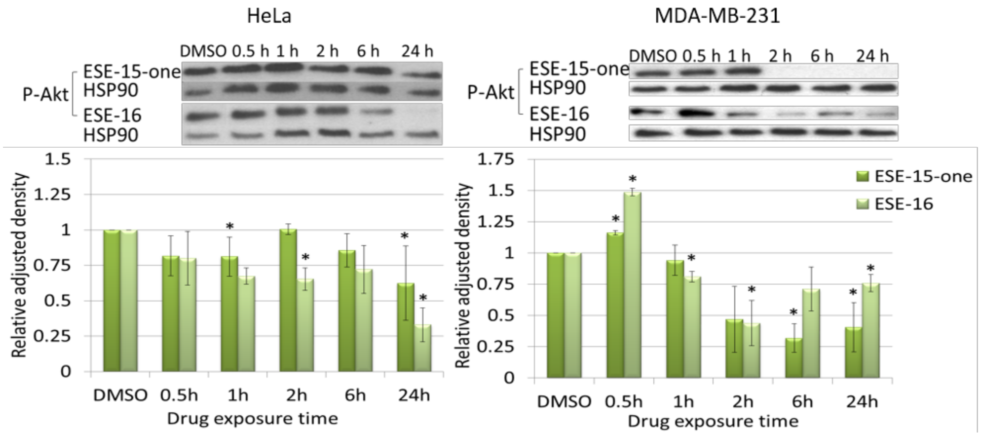

Figure S11. Sequential Akt phosphorylation in HeLa and MDA-MB-231 cells exposed to the 2-ME analogous. Bars represent a mean fold-increase of at least 3 repeats, the error bar show SEM, and *$p < 0.05$.

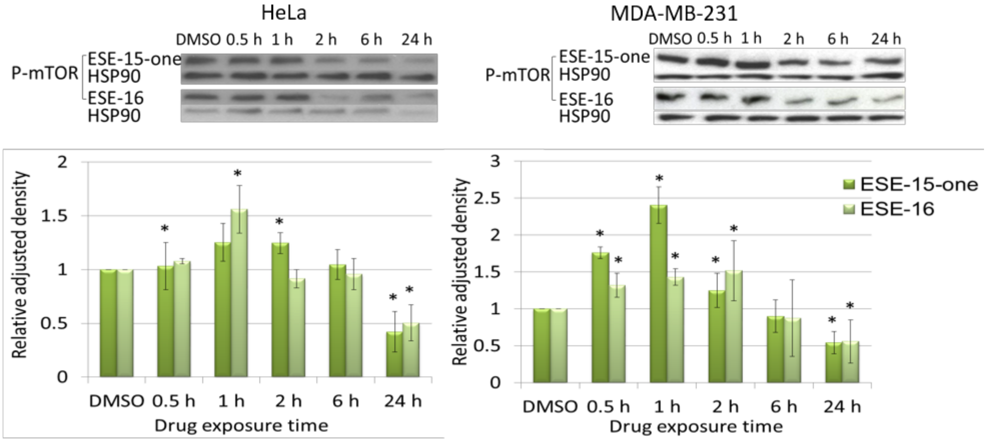

Figure S12. Sequential mTOR phosphorylation in HeLa and MDA-MB-231 cells exposed to the 2-ME analogous. Bars represent a mean fold-increase of at least 3 repeats, the error bar show SEM, and *$p < 0.05$.
