# Supplementary material for: Characterization of Signalling Pathways That Link Apoptosis and Autophagy to Cell Death Induced by Estrone Analogues Which Reversibly Depolymerize Microtubules
Source: Molecules. 2021 Jan 29;26(3):706. doi: 10.3390/molecules26030706 (PMC7866274; doi:10.3390/molecules26030706)
Supplement: Supplementary file 1 [file molecules-26-00706-s001.zip › Video S1 Time-Lapse Imaging.pptx]

## Slide 1
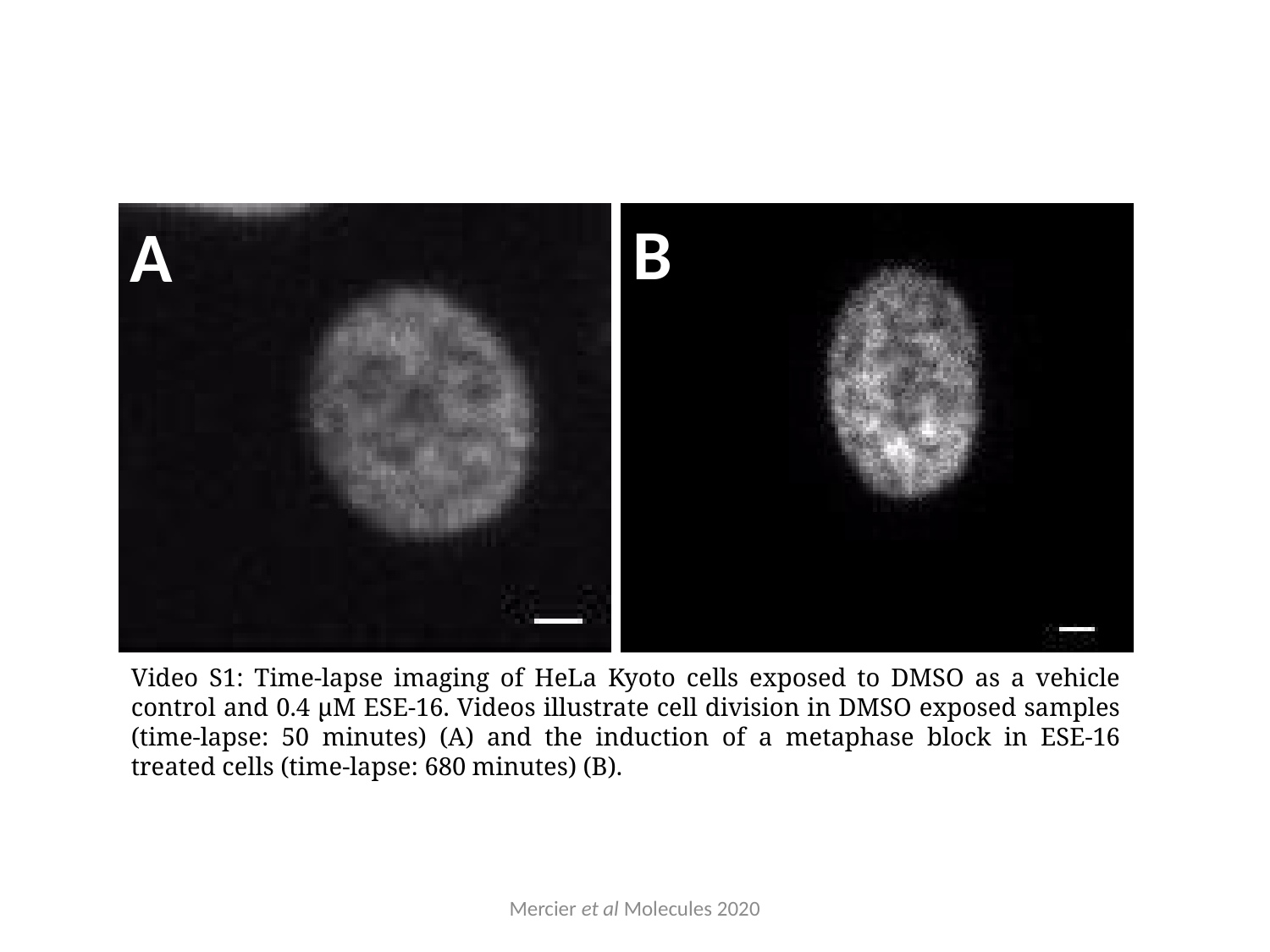

B
A
Video S1: Time-lapse imaging of HeLa Kyoto cells exposed to DMSO as a vehicle control and 0.4 µM ESE-16. Videos illustrate cell division in DMSO exposed samples (time-lapse: 50 minutes) (A) and the induction of a metaphase block in ESE-16 treated cells (time-lapse: 680 minutes) (B).
Mercier et al Molecules 2020
